# Identification of high-confidence RNA regulatory elements by combinatorial classification of RNA–protein binding sites

**DOI:** 10.1186/s13059-017-1298-8

**Published:** 2017-09-08

**Authors:** Yang Eric Li, Mu Xiao, Binbin Shi, Yu-Cheng T. Yang, Dong Wang, Fei Wang, Marco Marcia, Zhi John Lu

**Affiliations:** 1 MOE Key Laboratory of Bioinformatics, Center for Synthetic and Systems Biology, School of Life Sciences, Tsinghua University, Beijing, 100084 China; 2 Life Sciences Institute, Innovation Center for Cell Signaling Network, Zhejiang University, Hangzhou, Zhejiang 310058 China; 3 European Molecular Biology Laboratory, Grenoble Outstation, 71 Avenue des Martyrs, Grenoble, 38042 France

**Keywords:** RNA-binding protein, CLIP-seq, Non-negative matrix factorization, RBPgroup

## Abstract

Crosslinking immunoprecipitation sequencing (CLIP-seq) technologies have enabled researchers to characterize transcriptome-wide binding sites of RNA-binding protein (RBP) with high resolution. We apply a soft-clustering method, RBPgroup, to various CLIP-seq datasets to group together RBPs that specifically bind the same RNA sites. Such combinatorial clustering of RBPs helps interpret CLIP-seq data and suggests functional RNA regulatory elements. Furthermore, we validate two RBP–RBP interactions in cell lines. Our approach links proteins and RNA motifs known to possess similar biochemical and cellular properties and can, when used in conjunction with additional experimental data, identify high-confidence RBP groups and their associated RNA regulatory elements.

**Electronic supplementary material:**

The online version of this article (doi:10.1186/s13059-017-1298-8) contains supplementary material, which is available to authorized users.

## Background

RNA-binding proteins (RBPs) are essential to sustain fundamental cellular functions, such as splicing, polyadenylation, transport, translation, and degradation of RNA transcripts [1, 2]. One study estimated that more than 1500 different RBPs exist in human [3]. These RBPs cooperate or compete with each other in binding their RNA targets [4–6]. Many RBPs are capable of binding different RNA targets, partially by associating with different co-factors [7–9]. At the same time, some consensus RNA sequence motifs are recognized by homologous RBPs or homologous domains [10]. Thus, proteins and RNAs appear to interact in a combinatorial manner [11].

Recently, the advent of crosslinking immunoprecipitation sequencing (CLIP-seq) technologies, which combine immunoprecipitation with RNA–protein crosslinking followed by high-throughput sequencing, has enabled researchers to characterize transcriptome-wide RNA–protein binding sites with high resolution in different mammalian cells [12–14]. The CLIP-seq data of multiple RNA binding proteins have been curated and annotated in specific databases, such as CLIPdb, POSTAR, and STARbase [15–17]. Several significant studies improved the prediction of individual RBPs’ binding sites by training on CLIP-seq and RNAcompete datasets [18–20]. Systematic assessment of combinatory regulation of multiple RBPs would be more beneficial to derive precious biological information from various high-throughput CLIP-seq data.

Therefore, we analyzed the CLIP-seq data to group together RBPs that bind on the same RNA sites, in which proteins interact with RNAs in a combinatorial manner. For our classification, we used a soft-clustering method, non-negative matrix factorization (NMF), which allows each RBP to be clustered into more than one group, as it is the case for proteins that participate in multiple metabolic pathways. Using other RBPs’ binding signals as background also enables us to identify specific binding. Through our approach, we defined RNA–protein binding sites for the targeted RNAs and classified the corresponding RBPs into groups. Subsequently, we demonstrated that the binding sites we defined from RBP groups were supported by other types of biological data, such as alternative splicing (AS) and RNA degradation data. Furthermore, we compiled a web-based platform (http://RNAtarget.ncrnalab.org/RBPgroup) to make the binding sequences and enriched motifs easily accessible by the scientific community.

## Results

### Curation of various CLIP-seq data

**Fig. 1:**
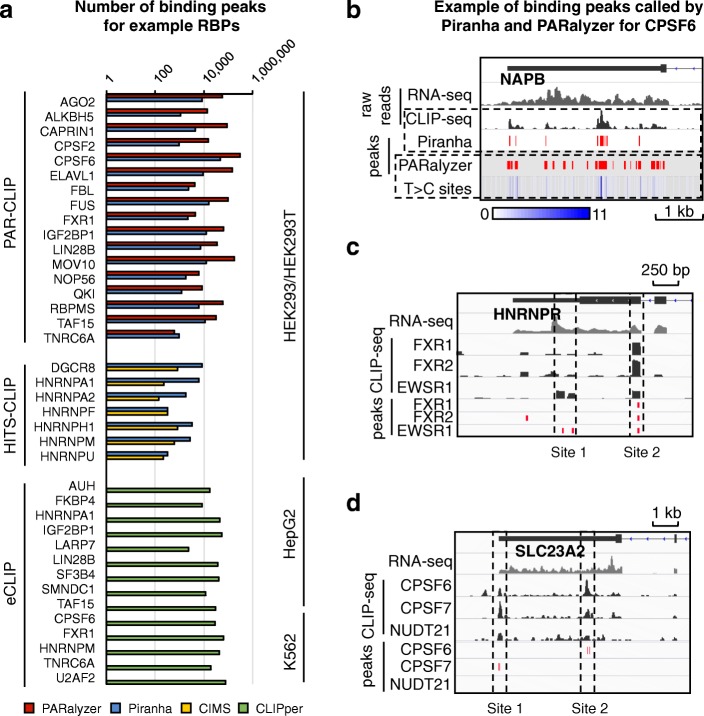
CLIP-seq data for different RBPs, CLIP technologies, and peak calling methods. **a** We show the numbers (log scale) of binding peaks defined by different peak calling methods for example RBPs. **b** CLIP-seq signals of an example RBP, CPSF6, binding on a gene, NAPB. The binding peaks are defined by Piranha (from signal height) and PARalyzer (from T to C mutation), respectively. **c**, **d** Examples of co-binding sites and binding peaks of single RBP. We show the raw reads and peaks identified by Piranha (*red bar*)

We collected 327 CLIP-seq datasets generated from three technical approaches: PAR-CLIP, HITS-CLIP, and eCLIP. The binding peaks of PAR-CLIP data were defined by Piranha [21] and PARalyzer [22]; the binding peaks of HITS-CLIP data were defined by Piranha and CIMS [23] (see detail in “Methods”). We also downloaded the binding peaks of eCLIP data, defined by CLIPper [24], from the ENCODE data portal (https://www.encodeproject.org). First, we can see that different RBPs display a very different number of binding peaks, ranging broadly from several thousands, i.e. for HNRNPU and TNRC6A, to tens of thousands, i.e. for CPSF6 and MOV10 (Fig. 1a, Additional file 1: Figure S1a). Such broad variance in the number of CLIP-seq peaks associated to each RBP is probably caused by many factors, such as differences in biochemical properties of the corresponding RBPs and in different labs’ experimental protocols. Second, our analysis shows discrepancies in the number of peaks obtained from different CLIP technologies and different peak calling methods (Additional file 1: Figure S1b). We show the CLIP-seq signal of an example RBP, where the binding peaks defined by two computational tools (Piranha and PARalyzer) are very different because they rely on two experimental features: Piranha defines binding peaks based on reads abundance, while PARalyzer utilizes the information of T to C mutation in PAR-CLIP (Fig. 1b, Additional file 1: Figure S2). Besides, yielding different number of peaks for each RBP, peak calling software also differ in terms of peak width. While Piranha and CIMS typically yield peaks of fixed width due to computational strategies being used [21, 23] (see detail in “Methods”), PARalyzer and CLIPper yield peaks of different length distributions (10–50 nt long for PARalyzer and 1–90 nt long for CLIPper) (Additional file 1: Figure S3).

In summary, deposited CLIP-seq datasets display significant variety probably due to intrinsic biochemical properties of RBPs, to differences in the experimental procedures and to the software used to identify RNA–protein interaction sites. These observations reflect the need to make CLIP-seq data analysis uniform and comparable and to define RNA–protein binding sites confidently and consistently. In this study, we aim to define a set of binding sites. We would sacrifice sensitivity and completeness to improve the accuracy of our prediction. Therefore, we only use peaks identified by multiple methods (e.g. PARalyzer and Piranha) (see detail in “Methods”).

### Clustering RNA–protein binding sites potentially identifies high-confidence interactions

We reason that a strategy to find high-confidence RNA–protein interactions in CLIP-seq data could be to identify RNA sites that are simultaneously associated with multiple RBPs. If the same RNA site shows CLIP signal for multiple RBPs that are biologically related, i.e. as part of the same macromolecular complex or as competitors on the same metabolic pathway, then such signal should be considered reliable. Two examples illustrate this idea. First, FXR1, FXR2, and EWSR1 have physical interactions between each other to form a complex and bind on the same RNA sites [25, 26]. When we align the CLIP-seq signals of these three RBPs together, a co-binding site shared by all three RBPs can be clearly recognized (site 2 in Fig. 1c), while the other one is probably ambiguous (site 1 in Fig. 1c). The second example consists of proteins NUDT21, CPSF6, and CPSF7, which usually bind on the same RNA regions for 3′-end polyadenylation of messenger RNA (mRNA)[27]. However, the peaks (binding sites defined by peak caller) cannot always be identified simultaneously from individual RBPs (sites 1 and 2 in Fig. 1d). Still, a weak binding signal missed for certain RBPs could be rescued by the strong signals of other “co-binding” RBPs if a method considers them together. These examples indicate that analyzing correlated RNA binding signals of multiple RBPs provides more information and potentially higher confidence than analyzing only the binding signal of individual RBPs.

### Unifying multiple RBP binding sites and binding affinities

**Fig. 2:**
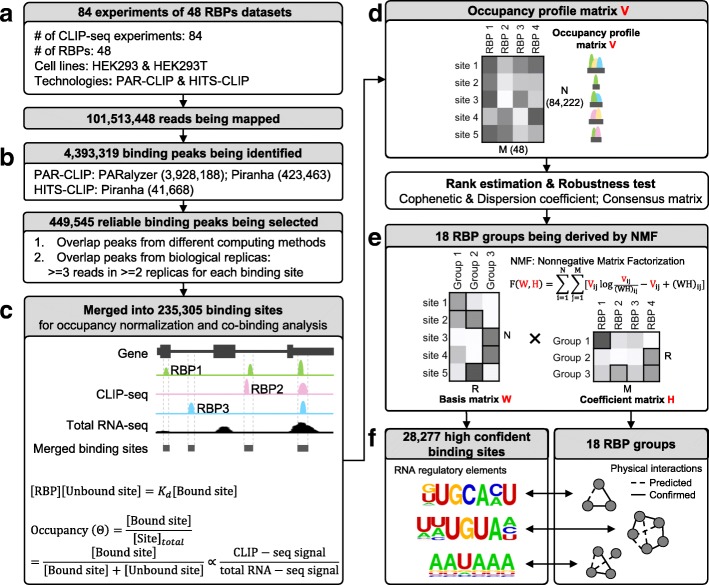
Integrative analytical pipeline for defining high-confidence RNA sequences/motifs bound by RBP groups. **a** In total, 84 CLIP-seq (including PAR-CLIP and HITS-CLIP) datasets of 48 human RBPs from HEK293/HEK293T cell lines were collected. **b** Different computing methods (e.g. Piranha and PARalyzer) were used to call peaks from raw reads for each RBP. Peaks from different methods and biological replicas were overlapped. **c** Then, the binding sites of all 48 RBPs were merged into one set of binding sites. RNA-sequencing (RNA-seq) data from corresponding cell lines were used to normalize the occupancy of each binding site. **d** Subsequently, an occupancy profile matrix V (N × M) was generated, representing the binding affinity for each binding site (*row*) bound by each RBP (*column*). **e** The occupancy profile matrix V was decomposed to a basis matrix W (N × R) and a coefficient matrix H (R × M). N denotes the number of binding sites; M denotes the number of RBPs; R denotes the number of groups. **f** The coefficient matrix was used to define the RBP components and their weights in each group. The basis matrix was used to define group-related binding sites (motifs) and binding affinities.

Based on the above observations, we set out to develop a systematic framework to define high-confidence RNA–protein binding sites in available CLIP-seq datasets (Fig. 2). To develop our method, we used 84 raw CLIP-seq datasets of 48 human RBPs [15, 16] obtained in HEK293/HEK293T cells (Fig. 2a, Additional file 1: Table S1).

We first filtered 450 K peaks out of ~4 M total peaks, retaining those that are reproducibly identified by multiple peak calling methods (PARalyzer and Piranha) and that occur in at least two biological replicas (Fig. 2b, see detail in “Methods”). We then merged these filtered peaks into a unified set of binding sites (235 K in total) (Fig. 2c). Among these merged binding sites, most were 20–60 nt in length, which corresponds to one- to twofold the length of non-merged binding peaks (Additional file 1: Figures S3, S4). Only ~10% of the merged binding sites were longer than 100 nt. We split these longer sites into 100-nt bins with 50-nt overlap for the following analyses.

Next, we generated an occupancy profile matrix from the merged binding sites (Fig. 2d). We used the CLIP-seq signals normalized by total RNA-seq signals obtained in HEK293 cell line as the values of our matrix (see detail in “Methods”). We discarded binding sites with no RNA-seq signals and those associated to only one RBP. This filtering procedure resulted in 84,222 binding sites used for clustering. The final occupancy profile matrix V is composed of N rows and M columns, where N is the number of filtered binding sites (84,222) and M is the number of RBPs (48).

### Cluster RNA–protein binding sites with non-negative matrix factorization

**Fig. 3:**
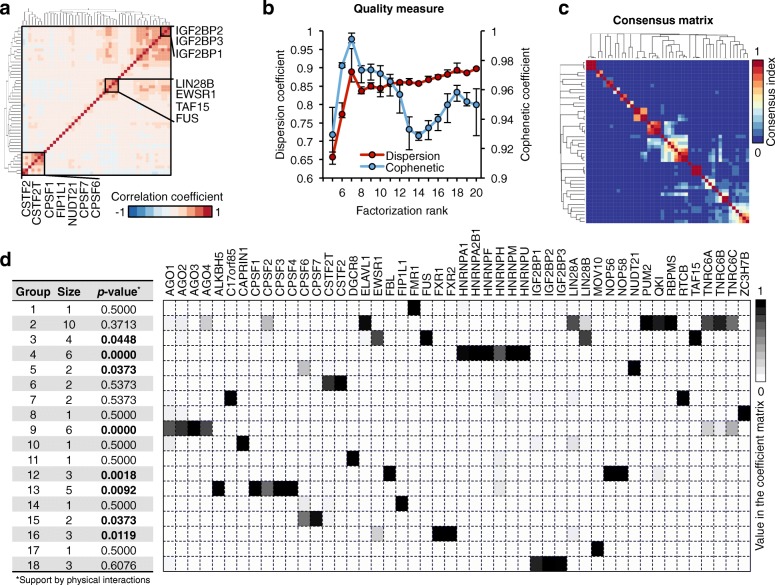
NMF inferred RBP groups from multiple RBPs. **a** Hierarchical clustering of the Spearman correlation coefficients between every two RBPs. **b** Criteria for estimating rank R in NMF. The cophenetic correlation coefficient (CPCC) and dispersion coefficient (DC) quantitatively measure the stability of clustering associated with each rank R, based on a consensus matrix. CPCC and DC scores are calculated in 10, 30, 50, 80, and 100 runs. Each point represents the mean value; up/down whisker represents the max/min value. **c** The average connectivity matrix over 30 factorization runs. **d** Eighteen RBP groups were inferred from 48 RBPs, supported by known physical interactions derived from GeneMANIA database. The *p* value was estimated from 10,000 random RBP sets for each group. *Heatmap* shows the weights of each RBP in each group, which were derived from the coefficient matrix. The values of each column are scaled (0 – 1)

On this unified occupancy profile matrix V (N × M), we applied a NMF method [28] (Fig. 2e) to identify groups of RBPs that bind on the same RNA sites (Fig. 2f). NMF has been used successfully in several biological applications [28–33] because of its non-negativity constraint and soft-clustering approach. Soft clustering allows one RBP to be clustered into multiple groups, which is needed in our case because many RBPs play multiple biological roles by interacting with different co-factors. As a comparison, we also calculated Spearman correlations between RBPs and grouped them with a hard-clustering hierarchical method. Although a few RBPs were grouped together as expected (e.g. IGBF2BP1, 2, and 3), many known complexes were not clustered well (e.g. HNRNPs) (Fig. 3a, Additional file 1: Figure S9a).

Decomposing the occupancy profile matrix V (N × M) using NMF, we derived the coefficient matrix H (R × M) (Fig. 2e), where R indicates the “rank,” i.e. the number of RBP groups. The key issue to decompose the occupancy profile matrix with NMF is to find a reasonable value for the rank R. We used cophenetic and dispersion correlation coefficients (Fig. 3b) to quantitatively measure the clustering stability associated with each rank R, based on a consensus matrix that is defined as the average connectivity matrix over multiple factorization runs [33] (see detail in “Methods”). We selected the local maxima 18 as the potential optimized value for R, from which we calculated the consensus matrix visualizing the robustness of our clustering (Fig. 3c). Thus, from the coefficient matrix, we identified R = 18 different RBP groups, such that in each group all RBPs bind on the same RNA sites (see detail in “Methods”). Twelve groups include more than one RBP (Fig. 3d). These RBPs correspond either to RBPs that compete for the same binding site on target RNAs or to different subunits of the same RNA-binding complex.

Moreover, by the basis matrix W (N × R) (Fig. 2e), we determined the number of all RNA sequences bound by each RBP group. In this step, other RBPs’ binding signals are treated as background for each group, which makes the identified binding sites more specific (see detail in “Methods”). In total, we identified 28,277 RNA sequences out of the ~4 M binding peaks initially identified in the raw PAR-CLIP datasets (Fig. 2b). Among these 28,277 RNA sequences, various consensus RNA motifs emerge and are enriched in each given group (see details later). An exhaustive compilation of all RBP groups, RNA sequences, and enriched motifs is hosted on our web-based platform (http://RNAtarget.ncrnalab.org/RBPgroup).

### RBPs grouped by NMF are supported by other types of associations

Having established a classification of RBPs by NMF clustering, we proceeded by analyzing the properties of various RBP groups in detail.

Among the 12 RBP groups that include more than one RBP, we found that eight groups were enriched with proteins that are known to interact with each other physically [34]. For example, the physical interactions of AGO protein and TNRC6A/C, which were clustered together as group 9, have been validated previously by affinity capture-Western experiment [35]. In these eight groups, the frequency by which known interactors fall into the same RBP group is significantly higher than the expectation from a randomized set of proteins (Fig. 3d, Additional file 1: Figure S5).

Besides grouping together known physical interactors, our clustering also groups together proteins that are known to be co-expressed, proteins that share domain similarity, proteins that participate in the same metabolic pathway, and proteins whose genes are related (Additional file 1: Table S2). For instance, group 18 contains IGF2BP family proteins, which share a protein domain, and group 7 contains C17orf85 and RTCB, which are related at the genetic interaction level.

Finally, considering that a distinctive feature of our soft-clustering NMF method is the ability to assign each RBP to multiple groups if they interact with different RNA targets, we looked for such “promiscuous” RBPs in our groups. We identified six RBPs in more than one group (Fig. 3d), namely AGO4 (in groups 2 and 9), CPSF2 (in groups 2 and 13), CPSF6 (in group 5, 14, and 15), EWSR1 (in groups 3 and 16), TNRC6A (in groups 2 and 9), and TNRC6C (in groups 2 and 9).

### NMF identifies significantly enriched RNA motifs associated to specific RBP groups

Besides grouping together RBPs that are biologically related, our NMF clustering pipeline also yields interesting information about the RNA binding motifs associated to such RBP groups.

**Fig. 4:**
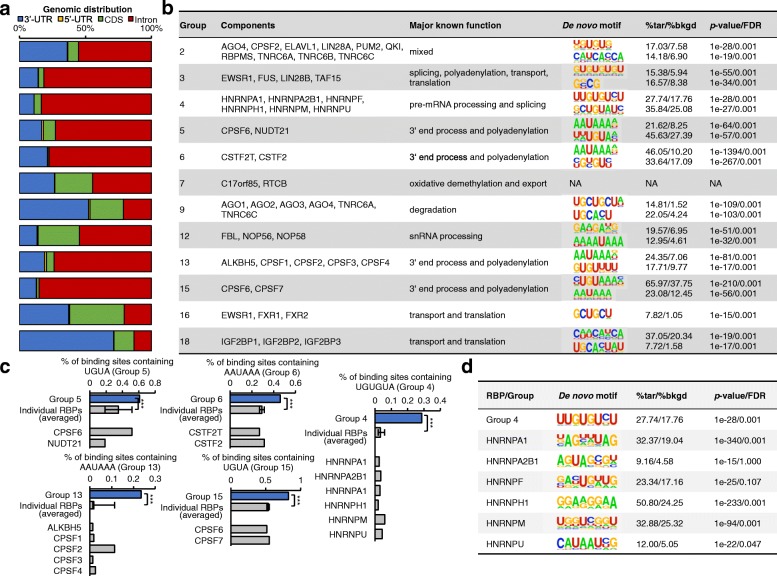
Enriched RNA motifs related to the RBP groups. **a** The histogram shows the genomic distribution of group-related binding sites for each group (with at least two RBP components). **b** De novo sequence motifs and known functions reported in literatures. The *p* values of the motifs were calculated by binomial test against the randomly selected genome background. The false discovery rate (FDR) was calculated using HOMER (option “–fdr 1000,” so the smallest number is 0.001). **c** Percentages of known motifs in the binding sites co-bound by each RBP group and in the binding peaks identified from individual RBPs. The percentage number is the portion of binding sites that contain the named motif (e.g. UGUA or AAUAAA). The average and standard deviation of the percentages for individual RBPs are also shown. The binding peaks of single RBP are the overlapped ones identified by both Piranha and PARalyzer using default parameters. The enrichment was tested with Fisher’s exact test between the group and individual RBPs. Four numbers were used in the test for each motif, total number of binding sites associated to the group, total number of binding peaks associated to an individual RBP, total number of binding sites and binding peaks containing the known motif. (***p value < 0.001) **d** We also predicted the de novo motifs (top one is shown for each) using HOMER from the binding sequences of group 4 and individual RBPs

First, the genomic distributions of the binding sites associated to many RBP groups are, in general, consistent with known functions of the corresponding RBPs (Fig. 4a, b). For instance, the RNAs associated to RBP group 4, which contains RBPs involved in splicing [36], are enriched in binding sites that are located in intronic regions. The RNAs associated to Group 5, 6, 13, and 15, which contain RBPs involved in polyadenylation [27, 37], are mainly located in 3′-UTR and intronic regions. Consistently, it was reported that widespread mRNA polyadenylation events could happen in introns, indicating dynamic interplay between polyadenylation and splicing [37]. Moreover, the RNAs associated to groups 9 and 18, which contain RBPs involved in mRNA degradation, mRNA transport, and translation [13], are enriched in binding sites that are located at 3′-UTR regions.

Second, we could identify similarities in the sequences of the binding sites associated to each RBP group. Such similarities allowed us to derive consensus RNA motifs that are particularly recurrent (enriched) for each RBP group (Fig. 4b, Additional file 1: Figure S6, see detail in “Methods”), in different genomic regions (i.e. 5′ UTR, 3′ UTR, CDS, and intron) (Additional file 1: Figure S6a). We note that the PAR-CLIP data could be biased by U content. To prevent the nucleotide bias, we extended the binding sites to upstream and downstream 100 nt for motif finding. Meanwhile, we used HOMER to normalize the control (background) to the same nucleotide content as the binding sites’ sequences. Each motif enrichment was calculated based on this normalized background (Additional file 1: Table S5). Some of the recurrent consensus motifs correspond to known protein-binding RNA motifs (Fig. 4c). For example, consensus motifs related to RNA polyadenylation, i.e. AAUAAA (polyadenylation signal [PAS]) and U-/GU- (downstream sequence element [DSE]), are recurrent among the binding sites associated to RBP groups 13 and 6, which contain proteins involved in RNA polyadenylation [38], i.e. cleavage and polyadenylation specificity factors (CPSFs) and cleavage stimulatory factors (CSTFs), respectively [39–41]. Moreover, motif UGUA, which is generally located upstream of 3′-end RNA cleavage sites [42, 43], is recurrent among the binding sites associated to RBP groups 5 and 15, which contain proteins involved in 3′-end cleavage of RNA transcripts, i.e. simplekin and cleavage factor Im (CFIm). Remarkably, the enrichment of these known motifs within the RBP groups identified by our clustering pipeline is statistically more significant than the occurrence of such motifs in the binding peaks identified from each single RBP (Fig. 4c). We also tested the enrichments with different thresholds of peak calling for individual RBPs and our observations were confirmed (Additional file 1: Figure S6b). We further compared the enrichment of known motifs in our RBP group-related RNA sequences with individual RBP-associated RNA sequences predicted by three published methods [18–20]. Our results showed substantially better enrichments than others (Additional file 1: Table S6). In addition to these known protein-binding motifs, we are also able to detect novel RBP association with other consensus motifs. For instance, motif UGUGU is a cis-regulatory element related to splicing [44] and de-novo finding detects a very similar motif as the most recurrent motif among binding sites associated to group 4 RBPs (binomial test, *p* value = 1E-28) (Fig. 4d). Group 4 contains six HNRNP splicing factors, which exclude exons and create sites of AS by interacting with silencer sequences [4, 45]. UGUGU occurs in ~30% of the binding sites associated to group 4, but only in 1–3% of the binding peaks associated to each single HNRNP. Such UGUGU motif could not have been predicted de novo from peak calling of individual HNRNPs (Fig. 4d). More novel consensus motifs are shown in a table (Fig. 4b).

All the above results show that the binding sites defined from our RBP groups are enriched with consensus motifs, including both known and novel motifs. Such binding redundancy and identification of enriched consensus motifs within RBP groups suggest that the binding sites filtered by our pipeline are specific, bona fide, high-confidence sites by protein interaction. To make these motifs easily accessed, we organized them in our web-based platform, available at http://RNAtarget.ncrnalab.org/RBPgroup.

### RNA binding sites associated to RBP groups are supported by other biological data

Having established that our clustering approach groups together proteins and RNA sequences that possess related biological functions, we questioned whether we could find other supporting evidence and correlations across our RBP groups or our RNA consensus motifs.

**Fig. 5:**
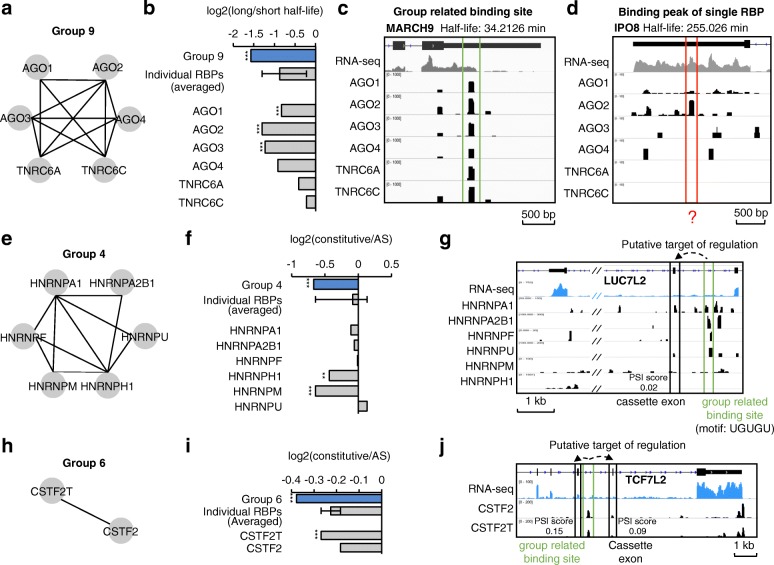
Representative RBP groups correlated with RNA degradation and splicing data. **a** Known physical interactions between RBPs in Group 9. **b** We calculated the fractions (log2 ratios) having binding sites co-bound by Group 9 (or binding peaks identified from individual RBP’s CLIP-seq data) for long half-life genes and short half-life time genes. The binding peaks of single RBP are the overlapped ones defined by both Piranha and PARalyzer using default parameters. The average and standard deviation of the ratios for individual RBPs are shown. The enrichment was tested with Fisher’s exact test for each individual RBP and the group, respectively. Four numbers were used in the test: total number of long half-life genes, total number of short half-life genes, numbers of long and short half-life genes containing the binding sites/peaks. (***p value < 0.001). **c** A group-related binding site located at 3′-UTR of gene MARCH9 with short half-life. **d** A single RBP’s binding site located at 3′-UTR of gene IPO8 with long half-life. **e**, **h** Known physical interactions between RBPs in groups 4 and 6. **f**, **i** We calculated the fractions (log2 ratios) having binding sites co-bound by RBPs in group 4/6 (or binding peaks identified from individual RBP’s CLIP-seq data) for AS exons and constitutive exons. The average and standard deviation of the ratios for individual RBPs are shown. The enrichment was tested with Fisher’s exact test for each individual RBP and the group, respectively. Four numbers were used in the test: total number of AS exons, total number of constitutive exons, numbers of AS and constitutive exons close to the binding sites/peaks. (**p value < 0.005; ***p value < 0.001). **g**, **j** Group 4/6 associated binding site, which is located near cassette exon(s) of gene LUC7L2/TCF7L2 with low PSI score. The binding site of group 4 contains the known motif, UGUGU

First, we noticed that RBP group 9 includes proteins AGO1-4 and TNRC6A/C (Fig. 5a), which function as a complex in miroRNA (miRNA)-mediated decay [46]. Therefore, we analyzed whether RNA binding sites associated to RBP group 9 have similar short half-lives. We downloaded the data of degradation rates (measured as half-lives time) of human mRNAs in HEK293 cell line from a previous study (GSE49831) [47]. We defined genes with half-life > 174 min (80th percentile of all genes) as long half-life genes, while genes with half-life < 40 min (20th percentile) were defined as short half-life genes. RNA binding sites co-bound by RBP group 9 are significantly enriched in short half-life genes (Fisher’s exact test, *p* value = 5.14E-11) (Fig. 5b). Such enrichment is higher among RNA binding sites associated to RBP group 9 than among the CLIP-seq peaks associated to each individual RBP (i.e. AGO1–4 or TNRC6A/C) (Fig. 5b). We also compared the enrichments under different thresholds of peak-calling for individual RBPs and the trend remained (Additional file 1: Figure S7a). As an example, we show a clear binding site associated to group 9 RBPs in MARCH9’s RNA, which has a short half-life (34 min) (Fig. 5c). Instead, RNA IPO8, which has a binding site only for AGO2, but not for other RBPs of group 9, is much more stable, with a half-life of 255 min (Fig. 5d). While AGOs and TNRCs thus likely bind on MARCH9 to degrade this RNA, AGO2 is unlikely to degrade IPO8’s RNA. Other metabolic functions of AGO2 may explain its interaction with IPO8, i.e. the role of AGO2 in translation inhibition. No other RBP group except group 9 includes RBPs involved in RNA decay. Consistently, when we examined the half-life of the RNAs associated to each of the other RBP groups, we did not find any significant correlations (Fig. 4b and Additional file 1: Figure S7b).

Second, we noticed that RBPs of groups 4 and 6 are strongly related to RNA splicing and cleavage [27, 36] (Fig. 4b). For example, group 4 contains six HNRNP splicing factors (Fig. 5e). Therefore, we examined if there is any correlation between RNA binding sites associated to RBP groups 4 and 6 and their splicing score (Additional file 1: Figure S8). A Percentage Spliced In (PSI) score was calculated for every exon using RNA-seq data from HEK293 cell line. We defined exons with PSI scores < 0.2 as AS exons, while exons with PSI > 0.8 as constitutive exons. As expected, the binding sites co-bound by group 4 are significantly closer to AS exons than to constitutive exons (Fisher’s exact test, *p* value = 4.88E-05) (Fig. 5f); and the enrichment is higher than the binding peaks identified from nearly all individual HNRNPs data and as good as HNRNPM data (Fig. 5f) independent of the thresholds used (Additional file 1: Figure S8b). Similarly, we found significant association between the splicing scores and binding sites co-bound by RBP group 6 (Fisher’s exact test, *p* value = 1.48E-04, Fig. 5i, Additional file 1: Figure S8c).

As mentioned above, the binding sites associated to group 4 RBPs are enriched with the UGUGU motif and are located close to transcript regions susceptible to AS (Fig. 4d). Here, we show an example of the binding on LUC7L2’s RNA, indicating proximity (~800–1000 nt distance) between a binding site with UGUGU motif that recruits splicing factors (HNRNPs) and their putative target splicing site (the cassette exon) (Fig. 5g). From the RNA-seq signals, we can see that the cassette exon is spliced (low PSI score) as a consistent result. Similarly, we show an example of the binding signals of RBPs in group 6 on TCF7L2’s RNA (Fig. 5j).

Such correlations between RNA sequences associated to the clustered RBP groups and their putative biological targets suggest that our clustering method has the potential to identify not only consensus protein-binding sequences in RNA but also functional RNA regulatory elements.

### NMF clustering can be applied to CLIP-seq data from any cell line and CLIP technology

**Fig. 6:**
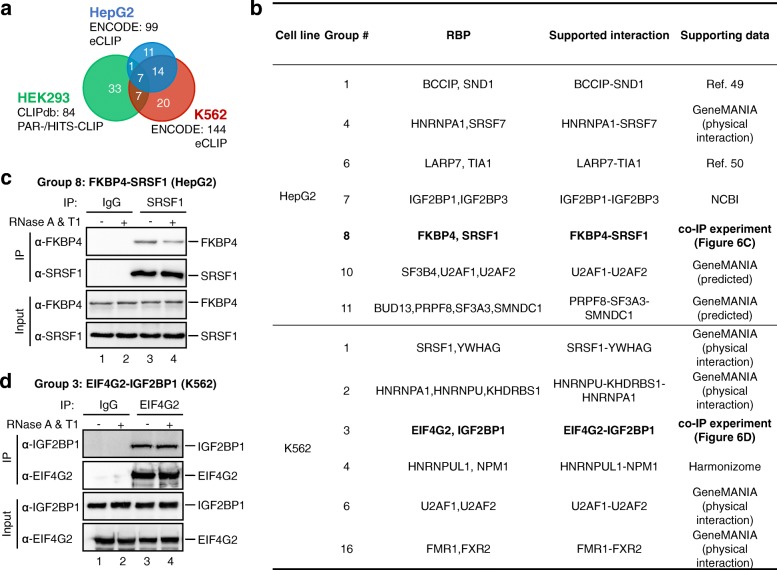
RBP groups inferred from eCLIP data and co-IP validation. **a** We show the summary of datasets for different CLIP technologies and cell lines used in this study. The *Venn diagram* shows only a few number of shared RBPs among three cell lines. **b** Example RBP groups having interactions supported by other data/evidence in HepG2 and K562 cell lines. **c** Co-IP of FKBP4 and SRSF1 (group 8) in HepG2 cell line. **d** Co-IP of EIF4G2 and IGF2BP1 (group 3) in K562 cell line

Our clustering approach could also be expanded to data obtained from other cell lines (i.e. HepG2 and K562) and CLIP methodologies (i.e. eCLIP). After using PAR-CLIP and HITS-CLIP data collected from different repositories to illustrate the advantage of our method, we further applied our method on eCLIP data generated by ENCODE [48]. ENCODE data have less heterogeneity than the PAR-CLIP and HITS-CLIP data because they were produced by the consortium using identical experimental procedures. In total, 99 eCLIP datasets of 33 RBPs in HepG2 cell line and 144 eCLIP datasets of 48 RBPs in K562 cell line were collected (Fig. 6a). First, we show that the RBPs are not able to be clustered well by hierarchical clustering methods (Additional file 1: Figure S9). Instead, using our soft-clustering method, the RBPs can be successfully clustered into 12 and 17 groups in HepG2 and K562 cell lines, respectively (Additional file 1: Tables S3, S4). Some of the groups, such as HNRNPA1-SRSF7 in HepG2 cell line, HNRNPA1-HNRNPU-KHDRBS1, U2AF1-U2AF2, and FMR1-FXR2 in K562 cell line, are significantly enriched with proteins that are known to physically interact. However, different from the result in HEK293/HEK293T data, the larger amount of RBPs in ENCODE generated a higher number of groups that are composed by proteins not known to interact together (Additional file 1: Tables S3, S4). Although not annotated as known physical interactions in the GeneMANIA database, several putative interactions were found to be reported by literatures or annotated as predicted interactions (Fig. 6b). For instance, BCCIP and SND1 in group 1 (HepG2 cell line) were reported to interact with MTDH [49]. IGF2BP1 and IGF2BP3 in group 7 (HepG2 cell line) were also supported by NCBI (https://www.ncbi.nlm.nih.gov/gene/10642), where LARP7 and TIA1 are proposed as candidates in regulating 5′ terminal oligopyrimidine (TOP) for mRNA translation [50]. HNRNPUL1 and NPM1 in group 4 (K562 cell line) were found to be supported by the Pathway Commons Protein-Protein Interactions dataset in Harmonizome database [51]. We also compared the RBP groups between these two cell lines (Additional file 1: Figure S10). A large proportion (17/21) of the shared RBPs were grouped with the other RBPs uniquely existing in different cell lines, which suggests different roles in different cell lines or data bias.

These groups represent interesting candidates for further exploration and discovery of potentially novel RNA–protein complexes. We experimentally validated the interactions in two putative groups, group 8 (FKBP4-SRSF1) in HepG2 cell line and group 3 (EIF4G2-IGF2BP1) in K562 cell line. SRSF1 is a splicing factor and FKBP4 is an immunophilin protein with PPIase and co-chaperone activities. Endogenous co-IP experiments were performed to assess FKBP4-RSF1 interaction in physiological condition. Endogenous FKBP4 could be detected in anti-SRSF1 IP complex, but not in control IgG (Fig. 6c). In addition, retrieved endogenous FKBP4 was obviously decreased after RNase A + T1 treatment (Fig. 6c, lane 4). These results suggest that SRSF1 is associated with FKBP4 in HepG2 in a RNA-dependent matter. The other validation experiment is the co-IP of two translational regulators, EIF4G2 and IGF2BP1, in group 3 (K562). Endogenous IGF2BP1 could be detected in anti-EIF4G2 IP complex, but not in control IgG (Fig. 6d, lane 4). RNase A + T1 treatment did not disrupt the interaction between EIF4G2 and IGF2BP1. These results suggest that EIF4G2 binds IGF2BP1 independent of RNAs in K562 cells.

## Discussion

Many of the published methods use CLIP-seq data as training data and aim to predict individual RBP’s binding sites [18–20]. Our method is different because we started from peaks defined from the CLIP-seq data. The focus and highlight of our study is the consequent results related to RBP groups: finding novel RBP–RBP associations, revealing enriched RNA motifs related to these RBP groups, and associating them with biological processes, such as AS and degradation.

We implemented a soft-clustering NMF approach to cluster protein-binding sites. We showed that the binding sites cannot always be clustered well by hierarchical clustering with three cut tree methods (i.e. dynamic tree, dynamic hybrid, and static) (Additional file 1: Figure S9). For instance, we identified 18 distinctive RBP groups using CLIP-seq data obtained from HEK293/HEK293T cell lines. However, the conventional methods (i.e. hierarchical clustering) only identified five clusters (Additional file 1: Figure S9), which are all included by the 18 clusters we identified. On the other hand, many well-supported known interactions were only detected by our method. For instance, the HNRNP, FBL-NOP56-NOP58, and AGO-TNRC6 groups are well-known interactions [13, 36, 52–54], which were successfully identified by our method, not the conventional clustering. Sometimes, two subunits of the same complex do not crosslink with the same efficiency. Our NMF method would also detect such binding site if the signal of one RBP is weak while the others are strong, because it considers the overall binding strength of the whole RBP group.

Our method has its limitations. We assume that overlap of peaks from proteins in the same pathways might indicate true binding sites. However, it could also be shared noise caused by the experimental or systematic bias. We would miss many single RBP-related binding sites because we only aim to find binding sites associated to RBP groups. We sacrificed the sensitivity and completeness to ensure the confidence of the final set. Some binding sites that cannot be detected by the current CLIP-seq experiments were also missed because our analyses did not predict new binding sites that cannot be identified from CLIP-seq data. In this paper, we used overlapped peaks defined by two computational tools (i.e. Piranha and PARalyzer); therefore, we would miss some true peaks identified by one method only. The accuracy of our results would also be significantly affected by false negatives caused by many other factors [55].

We found that our clustering approach could group together protein–RNA binding sites that have related biological functions. However, they could also be introduced by biological or technical artifacts. For instance, 41 out of 48 RBP data were produced by PAR-CLIP in HEK293/HEK293T, except for a few HITS-CLIP data for DGCR8, HNRNPA1, HNRNPA2B1, HNRNPF, HNRNPH1, HNRNPM, and HNRNPU. Using our method, HNRNPs were grouped together (Fig. 3d), which could be caused by either similar biological function of proteins in HNRNP family or technical batch effect. Still, among these HITS-CLIP RBPs, DGCR8 and HNRNPs were clustered into two different groups. In addition, we generated a new dataset consisting of different technologies to show that our method is able to cluster together RBPs in the same protein family without influence of technical bias (Additional file 1: Figure S11). We also noticed that RBPs characterized by different experimental studies could be clustered in the same group. For example, group 4 contains RBPs from two studies, GSE34996 [36] and GSE23694 [56], and group 9 contains RBPs from three studies, GSE43573 [52], GSE28865 [53], and GSE21578 [13]. These results suggest that our method is robust and can overcome the inconsistencies and heterogeneities related to individual sample processing and analysis.

We used the total RNA-seq data enriched from chromatin fraction to normalize the HEK293 dataset (see “Methods”), because most of the RBPs we studied in HEK293 are splicing and cleavage factors. According to the subcellular localization information of GeneCards (http://www.genecards.org/), almost all the RBPs we used in HEK293 exist in nucleus (Additional file 1: Table S1). Nevertheless, we recognized that using the total RNA-seq data from chromatin fraction may lead to some potential bias when normalizing the CLIP-seq signal. Therefore, we used 25 RBPs also confidently existing in cytoplasm and performed the normalization with a cytoplasm RNA-seq dataset (i.e. poly-A enriched RNA-seq, GSE68671) (Additional file 1: Figure S12). Seven groups (group c1–c7) were defined according to a coefficient matrix. Among the seven RBP groups, five of them (groups c1, c3, c4, c5, and c7) were overlapped with the previous results. Two of them (groups c1 and c5) were significantly supported by known physical interactions. Meanwhile, we calculated the fractions (log2 ratios) having binding sites co-bound by AGO proteins in Group c1 for long half-life genes and short half-life genes. RNA binding sites co-bound by RBP group c1 were significantly enriched in short half-life genes (Fisher’s exact test, *p* value = 4.64E-05) (Additional file 1: Figure S13). The conclusion is consistent with the result we got previously (Fig. 5b).

Furthermore, we determined that our clustering analysis suggests important correlations between the function of proteins that cluster in the same RBP groups. Finally, we identified a correlation between RNA sequences associated to our clustered RBP groups and their biological properties and/or their putative biological targets. These considerations suggest that our clustering method has the potential to identify not only consensus protein-binding sequences in RNA but also functional RNA regulatory elements. We will provide a useful tool for analyzing protein–RNA interaction datasets continuously being produced by CLIP-seq technologies and improving our understanding about the regulatory mechanisms of biologically important RNA–protein complexes.

## Conclusion

In summary, we show that integrating public CLIP-seq datasets can provide novel insights into the combinatorial classification of RBPs. We provide a unified and high-confidence set of protein-binding RNA sites and clustered RBP groups, which were validated by the known physical interactions and co-IP experiments. The binding sites defined by our method were more enriched with known motifs and better correlated with RNA degradation data and AS data than the binding sites of single RBPs. We shared our method and code, as well as the derived RNA regulatory elements, with the RNA community via a web-based platform (see “Availability of Data and Materials”).

## Methods

### Defining binding peaks from various CLIP-seq data

We collected 327 CLIP-seq datasets from the ENCODE data portal (https://www.encodeproject.org) and our published database, CLIPdb [15]. From ENCODE, we downloaded 99 and 144 eCLIP-seq datasets for 33 and 48 RBPs in HepG2 and K562 cell lines, respectively. The CLIP-seq data in CLIPdb [15] were collected from more heterogeneous resources. From CLIPdb, we used 84 CLIP-seq datasets in HEK293/HEK293T cell lines for 48 human RBPs. The experiments with large amounts of sequencing reads were preferred. The majority of data (41 out of 48 RBPs) are from photoactivatable ribonucleoside-enhanced crosslinking and immunoprecipitation (PAR-CLIP) [13]; the remaining data are from high throughput RNA sequencing and crosslinking (HITS-CLIP) [14] (Additional file 1: Table S1).

For ENCODE’s eCLIP data, we directly downloaded the binding peaks defined by CLIPper [24], with options –s hg19 –o –bonferroni –superlocal–threshold-method binomial–save-pickle, considering read 2 only (the read that is enriched for termination at the crosslink site) [48].

For PAR-CLIP data, we used two computational methods, Piranha [21] and PARalyzer [22], to identify the binding peaks of each RBP. Piranha identifies regions of statistically significantly larger read count than the background read-count distribution [21]. PARalyzer is designed for PAR-CLIP, which identifies RBP binding peaks by utilizing the distribution of thymine (T) to cytosine (C) transition in CLIP-seq read clusters [22]. By default, we used *p* value < 0.01 for Piranha and ModeScore > =0.5 for PARalyzer.

For HITS-CLIP data, we also used two computational methods, Piranha [21] and CIMS [23, 57], to identify the binding peaks of each RBP. The crosslinking-induced mutation site (CIMS) is designed for HITS-CLIP, which detects statistically significant mutations induced by protein–RNA crosslinking sites [23, 57]. Default parameters were used.

### Overlapping binding peaks of different peak calling methods and biological replicas

The quality of CLIP raw data largely depends on the complexity of library and depth of sequencing in each experiment. Therefore, different experiments, peak calling methods, and biological replicas for the same RBP could generate very different binding peaks [58]. We measured the similarity between replicas and different peak calling methods using the Jaccard similarity coefficient, which is defined as the number of regions that overlap between two peak sets, divided by the union of the two sets. The larger the Jaccard similarity coefficient, the more similar two peak sets are in terms of overlapping regions.

First, we overlapped the peaks identified by different methods for PAR-CLIP and HITS-CLIP data, because the identified binding peaks vary a lot among different peak calling methods. In general, PARalyzer identified more peaks than Piranha and CIMS found the least binding peaks. (1) For PAR-CLIP data, peaks variance is due to different conventions followed by different software to define an RNA–protein interaction peak. Piranha defines binding peaks based on reads abundance, while PARalyzer defines peaks based on the T to C mutations caused by the PAR-CLIP procedure. As a result, PARalyzer tends to generate more binding peaks than Piranha (Additional file 1: Figure S1, S2 and S14). We only kept the binding peaks identified by both PARalyzer and Piranha to ensure the confidence of our set from the very beginning. (2) For HITS-CLIP data, we kept the binding peaks defined by Piranha without considering CIMS, because CIMS identified too few peaks in our data (less than 200 for most cases). Notably, overlapping would miss some real binding sites only identified by one method, but it kept most confident ones (Fig. 2d). (3) For ENCODE’s eCLIP data, we used the downloaded peaks defined by one method, CLIPper [24].

Next, we adapted a previous method overlapping binding peaks identified from biological replicas [14]. Binding peaks with more than 3 reads in at least two replicas were kept. We used this semi-overlap strategy to save enough peaks, because the Jaccard similarities are usually low even for replicas of CLIP-seq data (Additional file 1: Figure S15).

We used the above overlapping steps to improve the data quality for the following analyses. Finally, we got three sets of binding peaks for different RBPs in three cell lines, HEK293/HEK293T, HepG2 (ENCODE), and K562 (ENCODE) (Additional file 1: Figure S16).

### Merging different binding peaks into one set of binding sites for multiple RBPs

To identify the combinatorial binding patterns of multiple RBPs, we merged the overlapping binding sites from all 48 RBPs into one set of merged binding sites on RNAs (Fig. 2c). The lengths of most binding sites were 20–60 nt, which is about one- to twofold of the average length of single RBPs’ binding sites (Additional file 1: Figures S3, S4). To reduce the bias from long binding sites in the downstream analysis, these long binding sites (length > 100 bp) were split into 100-bp bins with 50-bp overlap.

We annotated the binding sites to their located genomic regions (e.g. UTR, CDS, intron, miRNA, long non-coding RNA [lncRNA], etc) for each RBP, according to GENCODE (version 19) [59] and miRBase VX [60]. The annotation of each binding site was based on the following priority: CDS, canonical ncRNA, 3′-UTR, 5′-UTR, lncRNA exon, pseudogene, intron (mRNA and lncRNA), intergenic region, and others. The canonical ncRNAs include miRNA, small nuclear RNA, small nucleolar RNA, transfer RNA, ribosomal RNA, Y RNA, and 7SK RNA.

### Calculation and normalization of each RBP’s occupancy

The coverage of CLIP-seq reads in binding sites can be highly affected by the abundance of the transcripts [61]. Thus, we used total RNA-seq (GSE56862) by default in HEK293 and to normalize the binding affinity (i.e. occupancy [Θ]) of each merged binding site. We assume that the binding of RBP and a binding site is in equilibrium, which is based on the observation that the timescale of the binding and unbinding events (min) or the diffusion of the RBP (s) is much smaller than the half-life of most transcripts (h) [62]. Thus, the system reaches a steady state in a short time even when disturbances in the cell state (e.g. RBP concentration, transcript level) occurs. We also assume that the post-transcription regulation of the RBPs do not lead to major changes in the transcript levels. Then, the equilibrium equation is:

$$ \left[\mathrm{RBP}\right]\left[\mathrm{Unbound}\  \mathrm{site}\right]={K}_d\left[\mathrm{Bound}\  \mathrm{site}\right], $$

where [RBP], [Unbound site], [Bound site], and (K_d_) are the concentration of free RBPs, free RNA binding sites, RNA binding site being bound, and dissociation constant, respectively. Therefore, the occupancy, which is defined as the fraction of transcripts that is bound by an RBP at the binding site, is proportional to the CLIP-seq signal (RPM, read counts per million mapped reads) divided by total RNA-seq signal (for eCLIP, we used the input signal instead):

$$ \Theta =\frac{\left[ Bound site\right]}{{\left[ Site\right]}_{total}}=\frac{\left[ Bound site\right]}{\left[ Bound site\right]+\left[ Unbound site\right]}=\frac{\left[ Bound site\right]}{\left[ Bound site\right]+{K}_d\frac{\left[ Bound site\right]}{\left[ RBP\right]}} $$

$$ \propto \frac{\mathrm{normalized}\kern0.5em \mathrm{CLIP}-\mathrm{seq}\ \mathrm{signal}}{\mathrm{normalized}\  \mathrm{total}\ \mathrm{RNA}-\mathrm{seq}\ \mathrm{signal}}. $$

We scaled the ratio by the 95% quantile, which is a reasonable estimate for full occupancy under the assumption that the top 5% of binding sites are nearly fully occupied on each transcript and that crosslinking efficiency for a given factor does not depend strongly on sequence context. According to the definition, the occupancy is treated as a probability; therefore, the ratios for each RBP were scaled into the range of 0–1 [61].

Finally, we generated the occupancy profile matrix (Fig. 2d) for all merged binding sites (N rows) bound by different RBPs (M columns).

### Soft-clustering based on non-negative matrix factorization

NMF is a matrix factorization technique that can be applied to multidimensional data to reduce dimensionality. It usually approximately factorizes the original matrix into two matrices, with the constraint that all three matrices have no negative elements. This non-negativity makes the resulting matrices easier to be interpreted as biological meaningful features [33]. We adapted the NMF (R package: NMF 0.17.6) [63] method to decompose the occupancy profile matrix V (N × M, N rows: merged binding sites, M columns: RBPs) (Fig. 2e) into a coefficient matrix H (R × M) and a basis matrix W (N × R), with a given rank R:

$$ \mathrm{V}\approx \mathrm{WH}, $$

by minimizing the Kullback–Leibler distance between the original matrix V and WH:

$$ \mathrm{F}\left(\mathrm{W},\mathrm{H}\right)=\sum_{\mathrm{i}=1}^{\mathrm{N}}\sum_{\mathrm{j}=1}^{\mathrm{M}}\left[{\mathrm{V}}_{\mathrm{i}\mathrm{j}}\log \frac{{\mathrm{V}}_{\mathrm{i}\mathrm{j}}}{{\left(\mathrm{W}\mathrm{H}\right)}_{\mathrm{i}\mathrm{j}}}-{\mathrm{V}}_{\mathrm{i}\mathrm{j}}+{\left(\mathrm{W}\mathrm{H}\right)}_{\mathrm{i}\mathrm{j}}\right]. $$

The optimization can be solved by iterative updates of the matrix W and H. The basis matrix defines group-related binding sites, which can be used to associate the binding affinity with post-transcriptional regulatory events (e.g. AS, degradation) and identify putative motifs. The coefficient matrix defines the RBP components and their weights in each group.

The key issue to decompose the occupancy profile matrix is to find a reasonable value for the rank R (i.e. the number of RBP groups). Several criteria have been proposed to decide whether a given rank R decomposes the occupancy profile matrix into meaningful clusters. The cophenetic correlation coefficient (CPCC) and dispersion coefficient (DC) quantitatively measure the stability of clustering associated with a given rank R, based on a consensus matrix C (M × M) that is defined as the average connectivity matrix of cluster components (RBPs) over multiple factorization runs:

$$ \mathrm{C}\left(\mathrm{i},\mathrm{j}\right)=\frac{1}{\mathrm{Number}\_\mathrm{of}\_\mathrm{Runs}}{\sum}_{\mathrm{k}=1}^{\mathrm{Number}\_\mathrm{of}\_\mathrm{Runs}}{\mathrm{C}}_{\mathrm{k}}\left(\mathrm{i},\mathrm{j}\right), $$

$$ {\mathrm{C}}_{\mathrm{k}}\left(\mathrm{i},\mathrm{j}\right)=\left\{\begin{array}{cc}\hfill 1\hfill & \hfill \mathrm{if}\  \mathrm{items}\ \mathrm{i}\ \mathrm{and}\ \mathrm{j}\ \mathrm{belong}\  \mathrm{to}\  \mathrm{the}\  \mathrm{same}\  \mathrm{cluster}\hfill \\ {}\hfill 0\hfill & \hfill \mathrm{otherwise}\hfill \end{array}\right.. $$

The CPCC is a measure (ranges from –1 to 1) of how faithfully a dendrogram generated by a hierarchical clustering algorithm preserves the pairwise distances between the original un-modeled samples. Those distances are defined as a symmetric matrix D = (1-C), which is named dissimilarity matrix. The cophenetic distance is defined as the minimum dissimilarity level to merge two samples into the same cluster in a hierarchical clustering algorithm. Then the cophenetic distance between samples forms the cophenetic matrix T, which is also a symmetric matrix. Finally, the CPCC is computed as the Pearson correlation coefficient of the M(M-1)/2 upper diagonal elements of D and T:

$$ \mathrm{CPCC}=\frac{\sum_{\mathrm{i}<j}\left(\mathrm{D}\left(\mathrm{i},\mathrm{j}\right)-\overline{\mathrm{d}}\right)\left(\mathrm{T}\left(\mathrm{i},\mathrm{j}\right)-\overline{\mathrm{t}}\right)}{\sqrt{\left({\sum}_{\mathrm{i}<j}{\left(\mathrm{D}\left(\mathrm{i},\mathrm{j}\right)-\overline{\mathrm{d}}\right)}^2\right)\ \left({\sum}_{\mathrm{i}<j}{\left(\mathrm{T}\left(\mathrm{i},\mathrm{j}\right)-\overline{\mathrm{t}}\right)}^2\right)}}, $$

where $\overline{\mathrm{t}}$ and $\overline{\mathrm{d}}$ are the mean of M(M-1)/2 upper diagonal elements of T and D.

The DC reflects the dispersion (range of 0–1) of the consensus matrix C from the value 0.5. The closer to 1 is the DC, the more perfect is consensus matrix, and thus the more stable is the clustering. In a perfect consensus matrix, all entries are 0 or 1, meaning that all connectivity matrices are identical. The DC is defined as:

$$ \mathrm{DC}={\sum}_{i=1}^M{\sum}_{j=1}^M4{\left(C\left(i,j\right)-\frac{1}{2}\right)}^2. $$

We searched ranks from 5 to 20 and performed 10, 30, 50, 80, and 100 runs for each rank to find the local maximums of CPCC and DC as potential ranks (Fig. 3b, c). Subsequently, we used the potential ranks to decompose the occupancy matrix into basis matrix and coefficient matrix with 100 runs and then selected the decomposition result with the lowest approximation error (i.e. Kullback–Leibler distance) among the 100 runs. We also showed that the local maximum rank, 18, generated groups better supported by other evidence than the global maximum rank, 7 (Additional file 1: Figure S17).

### Identification of protein components in each RBP group

We used the coefficient matrix to define RBP groups. The RBPs in one group could probably compete with each other for the same binding site or form a complex to bind RNAs. In the coefficient matrix, each row represents one RBP group and each column represents one RBP. The values in the matrix indicate the weights of RBPs in their corresponding RBP group. The coefficient matrix was then scaled by column (RBP) to 0–1. Subsequently, we used a coefficient threshold 0.2 (~95th percentile of the whole matrix) to define the RBP components in each RBP group.

### Defining binding sites and their binding affinities associated to each RBP group

We associated each RBP group with binding sites according to the basis matrix. For each binding site and each group, we derived a basis coefficient score, which represents the binding affinity contributed by all RBPs in the defined group. In addition, we also calculated a basis-specificity score for each binding site using “kim” methods (*featureScore* in NMF package) [32]. The basis-specificity score is in the range of 0–1. The RBP group with a high basis-specificity score means that it specifically associates with the binding site. When the basis-specificity score is > 0.8 and the basis coefficient score is > 80% quantile for each column, we associate them together. In this way, our method rules out the unspecific binding associated with a large number of RBPs, which may be caused by high RNA abundance.

### Evidence used to validate the putative RBP groups

We used various protein–protein interactions in the GeneMANIA database [34] to confirm our predicted RBP groups (i.e. whether the RBPs within the predicted RBP group have known associations). GeneMANIA contains a large set of protein–protein association data with multiple types of evidence, including physical interactions, genetic interactions, pathways, co-expression, co-localization, and protein domain similarity. We downloaded all the protein–protein associations of 48 RBPs and calculated the association number for each RBP in each group by counting its connectivity with other RBPs. Then, we calculated the *p* value for each type of supporting evidence in each RBP group, based on the background of 10,000 random gene sets of the same size (Additional file 1: Figure S5).

### Motif analysis for the binding sequences of RNAs

De novo motif finding was performed using RNA sequences in individual RBP binding peaks and binding sites co-bound by RBPs in each group. HOMER tool was used for this analysis (findMotifsGenome.pl with parameters -len 4,6,8 -norevopp -rna) [64]. Background sequences were randomly selected from the genome, which matched the GC-content distribution of the input sequences. The *p* values were calculated by binomial test as default. In the main result, we showed the top two motifs with *p* values < 1E-10, with HOMER motif files provided in our supplementary website, http://RNAtarget.ncrnalab.org/RBPgroup. We searched the known motifs, which were derived from the literature [39, 44], within each set of binding sites/peaks using HOMER [64]. The enrichment was tested with Fisher’s exact test between the group and individual RBPs. Four numbers were used in the test for each known motif, total number of binding sites associated to the group, total number of binding peaks associated to an individual RBP, total number of binding sites, and binding peaks containing each known motif.

### Enrichment calculation for RNA degradation

We downloaded the degradation rates (measured as half-lives time) of human mRNAs in HEK293 cell line from a previous study (GSE49831) [47]. In total, 1626 genes with long half-life time (over 80th percentile; 174 min) and 1628 genes with short half-life time (below 20th percentile; 40 min) were selected. We then overlapped the binding sites/peaks with these genes using BEDTools [65]. The fraction of genes having the RBP (group) binding sites/peaks was calculated. The log2 ratio of the fractions for the long half-life genes and short half-life genes were shown in the main figure. The enrichment was tested with Fisher’s exact test for each RBP group and each individual RBP, respectively. Four numbers were used in the test for each set of binding peaks/sites: total number of long half-life genes, total number of short half-life genes, and numbers of long and short half-life genes containing the binding sites/peaks.

### Enrichment calculation for alternative splicing

We used the RNA-seq data in HEK293 (GSE44267) [66] to calculate PSI score, in the range of 0–1, with MISO tool [56]. A total of 10,713 exons with PSI score < 0.2 were regarded as AS exons; 9940 exons with PSI score > 0.8 were regarded as constitutive exons. Considering that splicing can be regulated by the flanking sequence of the exon, we associated the RBP (group) binding sites/peaks at a distance of 2 kb upstream and downstream of the exon, which would be most likely to affect splicing [36]. We calculated the fraction of exons having the RBP (group) binding sites/peaks. The log2 ratio of the fractions for AS exons and constitutive exons were shown in the main figure. The enrichment was tested with Fisher’s exact test for each RBP group and each individual RBP, respectively. Four numbers were used in the test for each set of binding peaks/sites: total number of AS exons; total number of constitutive exons; and numbers of AS and constitutive exons close to the binding sites/peaks.

### Cell lines and immunoprecipitation

HepG2 cells were grown in DMEM high glucose medium (Corning) with 10% fetal bovine serum (FBS) (GIBCO). K562 cells were maintained in RPMI 1640 (Corning) with 10% FBS. Endogenous Co-IP was carried out using indicated antibody and Protein A/G Magnetic Beads (Thermofisher, 88803). After RNase A/T1 Mix (Thermofisher, EN0551) treatment and several times of washes, precipitated protein were eluted in SDS loading buffer and separated by SDS-PAGE, transferred onto PVDF membranes (Millipore) and detected blots with appropriate antibodies. Antibodies used in this study include: anti-SRSF1 (Abcam, ab38017), anti-FKBP4 (Abcam, ab129097), anti-EIF4G2 (Cell Signaling, 5169), and anti-IGF2BP1 (Abcam, ab107205).

## Additional file

Additional file 1: Supplementary figures and tables. (PDF 1961 kb)
