# Supplementary material for: Identification of high-confidence RNA regulatory elements by combinatorial classification of RNA–protein binding sites
Source: Genome Biol. 2017 Sep 8;18:169. doi: 10.1186/s13059-017-1298-8 (PMC5591525; doi:10.1186/s13059-017-1298-8)
Supplement: Additional file 1: — Supplementary figures and tables. (PDF 1961 kb) [file 13059_2017_1298_MOESM1_ESM.pdf]

Additional file

Identification of high-confidence RNA regulatory elements by combinatorial classification of RNA-protein binding sites

Supplementary Figures..2
Figure S1 | Summary of datasets and the number of binding peaks....................................2
Figure S2 | The overlapping ratio between two peak sets called by Piranha and PARalyzer using different cutoffs..3
Figure S3 | Length distribution of each RBP's binding peaks..4
Figure S4 | Length distribution of the merged binding sites..5
Figure S5 | Randomization test for predicted RBP groups..5
Figure S6 | The regulatory elements (motifs) for each RBP group in different regions and percentage of known motif in binding sites/peaks...7
Figure S7 | The association between group related binding sites and degradation for each RBP group...8
Figure S8 | The association between group related binding sites and alternative splicing for each RBP group...9
Figure S9 | Hierarchical clustering of Spearman correlation coefficients between any two RBPs..11
Figure S10 | The comparison of RBP groups between HepG2 and K562 datasets.............12
Figure S11 | RBPs in the same family were clearly clustered together without influenced by technical bias..13
Figure S12 | NMF analysis for cytosolic RBPs...14
Figure S13 | The association between group related binding sites and RNA degradation for group c1...15
Figure S14 | Jaccard similarity between peak calling methods for three cell lines.............16
Figure S15 | Jaccard similarity between replicas for three cell lines..................................17
Figure S16 | The number of binding peaks selected for CLIP-seq data in three cell lines..18
Figure S17 | NMF analysis on low rank..19
Supplementary Tables..20
Table S1 | Human RNA-binding proteins collected for HEK293/HEK293T cell lines......20
Table S2 | The *p*-value of supporting evidence for each RBP group in HEK293/HEK293T cell lines...26
Table S3 | The *p*-value of supporting evidence for each RBP group in HepG2 cell line (ENCODE)...28
Table S4 | The *p*-value of supporting evidence for each RBP group in K562 cell line (ENCODE)...29
Table S5 | Nucleotide content in the sequences for motif identification............................31
Table S6 | The comparison of known motifs' enrichments in binding sites inferred by multiple methods..35
Supplementary References...39

Supplementary Figures

a

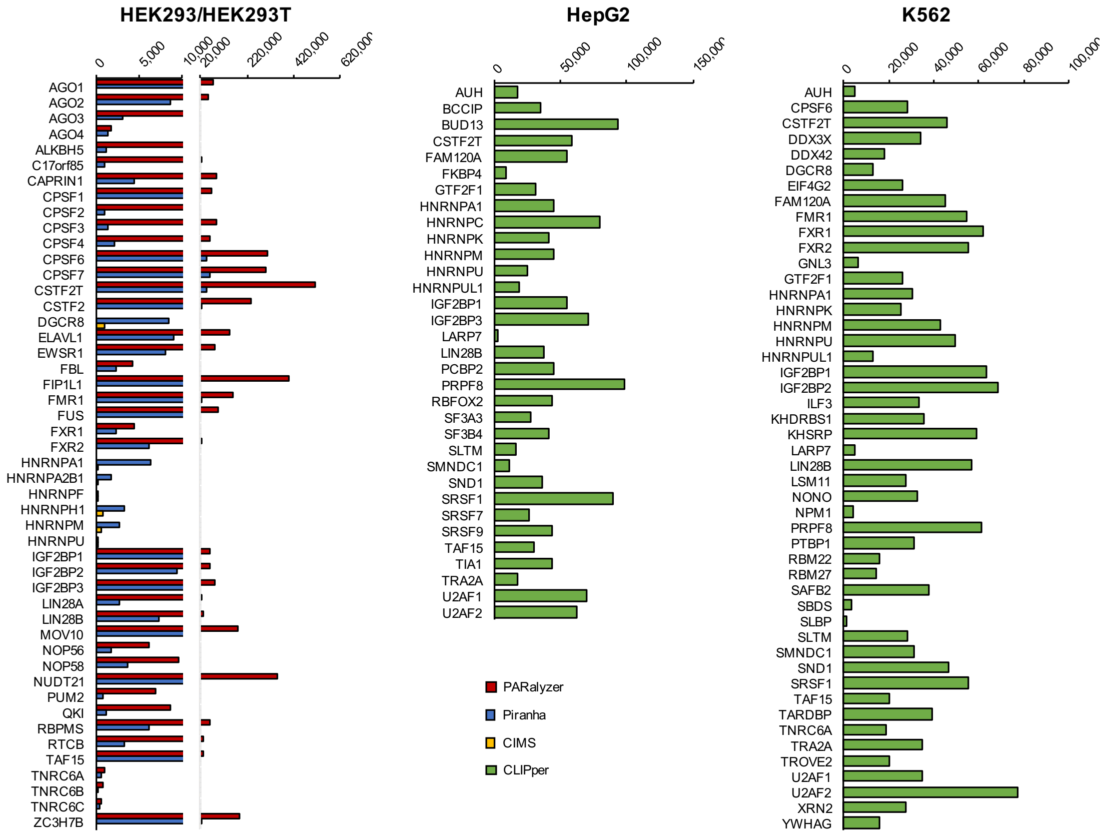

b

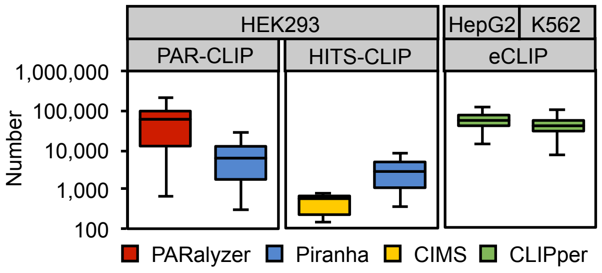

Figure S1 | Summary of datasets and the number of binding peaks

(a) The barplots show the number of binding peaks for each RBP dataset called by four peak calling methods in three cell lines. **(b)** The boxplot summarizes the numbers of binding peaks called by different peak calling methods for three CLIP-seq technical approaches.

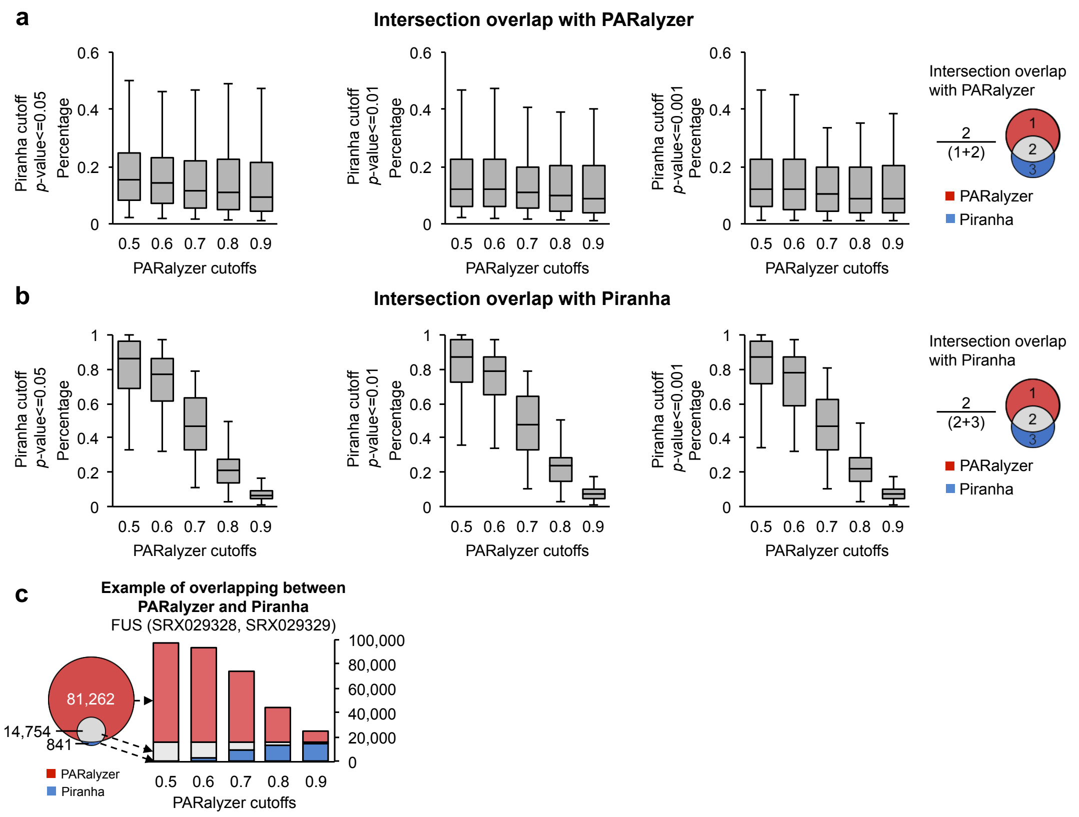

Figure S2 | The overlapping ratio between two peak sets called by Piranha and PARalyzer using different cutoffs

We have tried different threshold of Piranha (*p*-value smaller than 0.05, 0.01, 0.001, respectively) and PARalyer (the ModeScore from 0.5 to 0.9, which represents the "strength" of the signal from that cluster) to compare the identified peaks, we noticed that the more stringent threshold does generate less binding sites, as expected, but also less consistency (i.e. averaged overlapping ratio) in the total number of peaks called by PARalyzer **(a)** vs Piranha **(b)**. **(c)** For instance, Piranha identifies 47,500 peaks (*p*-value <= 0.01) for protein CPSF6, but PARalyzer (ModeScore >= 0.5) identifies 308,009 peaks with a small portion being overlapped. Using a more stringent threshold (i.e. ModeScore >= 0.9), the discrepancy between PARalyzer and Piranha cannot be solved (overlap even was decreased to 5% from 13%).

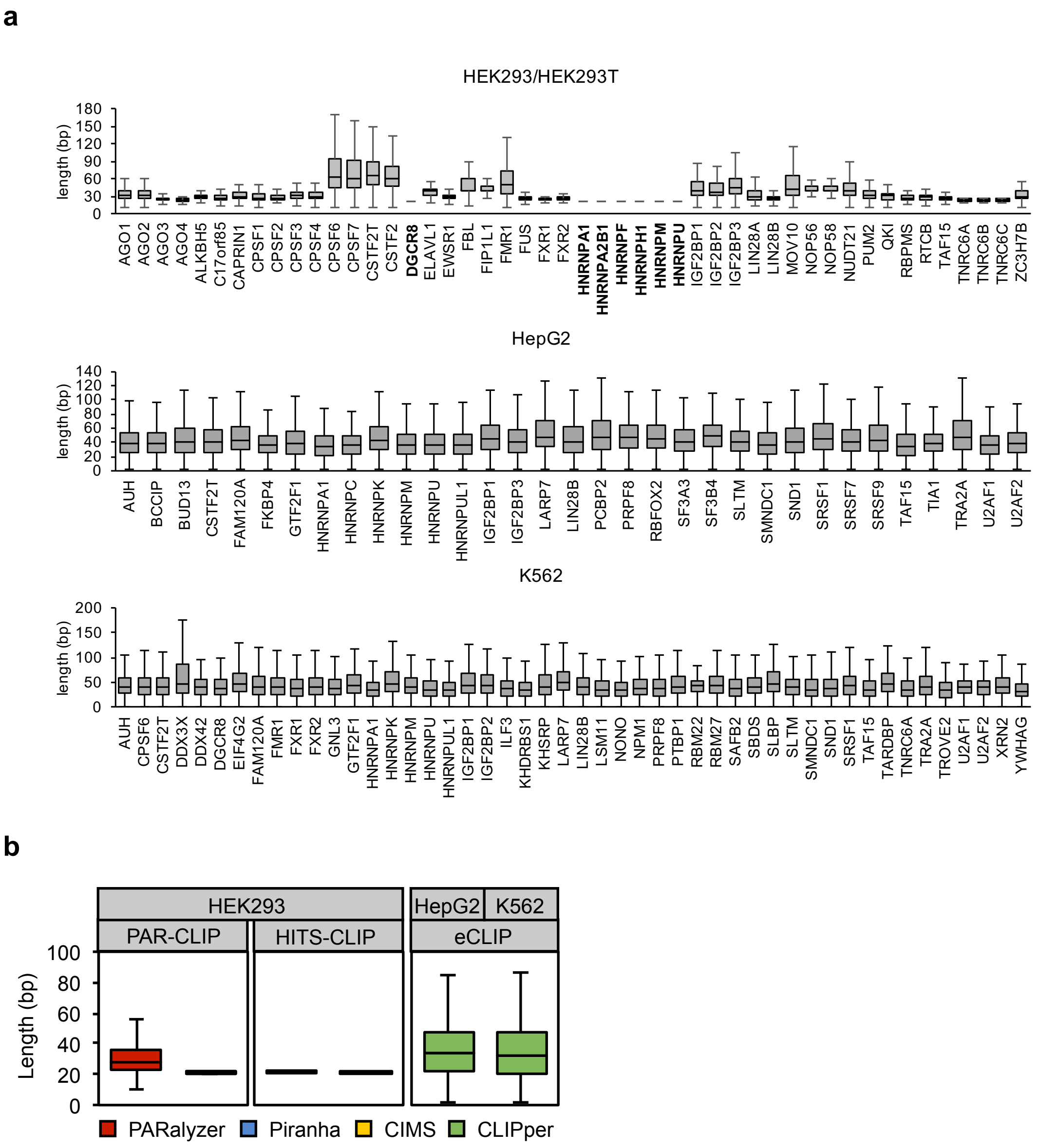

Figure S3 | Length distribution of each RBP's binding peaks

(a) Boxplot shows the average length of binding peaks for individual RBP in three cell lines. In HEK293/HEK293T cell line, the binding sites of most PAR-CLIP datasets (normal font) were called by Piranha (p-value<0.01) and PARalyzer (ModeScore>=0.5). The binding sites for HITS-CLIP datasets (bold font) were called by Piranha (p-value<0.01) only. In HepG2 and K562 cell line, the binding sites of eCLIP data were called by CLIPper from ENCODE website. **(b)** The boxplot summarizes the lengths of binding peaks.

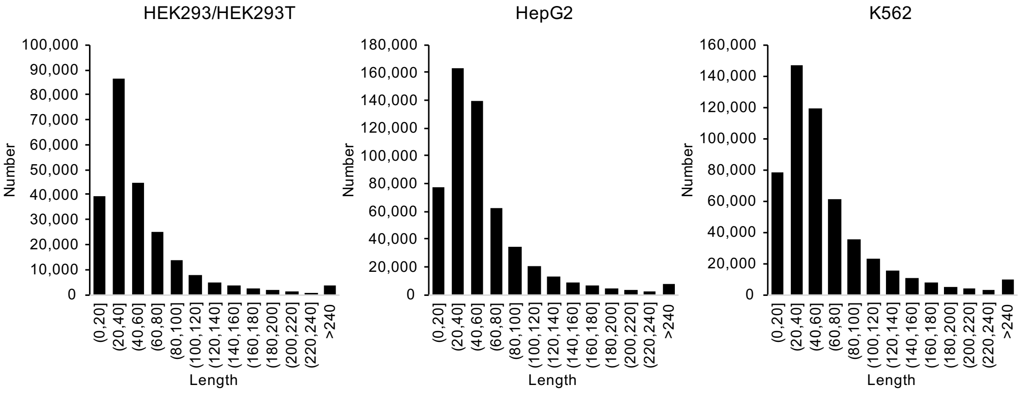

Figure S4 | Length distribution of the merged binding sites

The length distribution of merged binding sites in three cell lines.

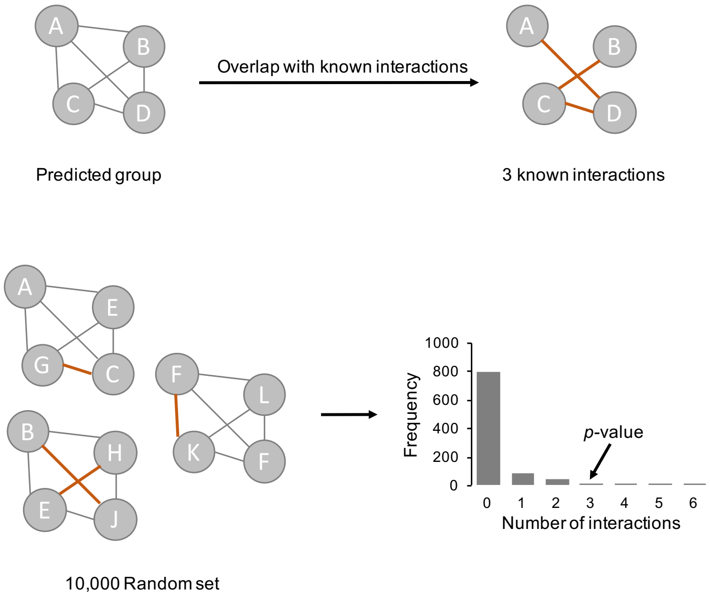

Figure S5 | Randomization test for predicted RBP groups

For each predicted RBP group, each pair of association is overlapped with the known associations derived from GeneMANIA database [1]. 10,000 random sets with the same size are used to obtain the frequency distribution of validated (overlapped) associations in a certain RBP group. The *p*-value is defined as the cumulative frequency at the right side.

a

Group	3'-UTR			5'-UTR			CDS			Intron		
	de novo motif	%tar/%bkgd	p-value/FDR	*de novo* motif	%tar/%bkgd	p-value/FDR	*de novo* motif	%tar/%bkgd	p-value/FDR	*de novo* motif	%tar/%bkgd	p-value/FDR
2	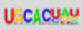	11.21/1.02	1e-36/0.001	NA	NA	NA	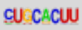	15.74/1.25	1e-13/0.001	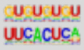	19.56/8.84<br>17.22/7.79	1e-18/0.001<br>1e-16/0.001
3	NA	NA	NA	NA	NA	NA	NA	NA	NA	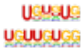	17.00/5.40<br>14.78/5.25	1e-69/0.001<br>1e-50/0.001
4	NA	NA	NA	NA	NA	NA	NA	NA	NA	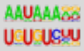	17.32/6.17<br>17.83/8.07	1e-58/0.001<br>1e-39/0.001
5	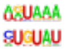	39.18/18.45<br>58.42/36.92	1e-15/0.001<br>1e-13/0.001	NA	NA	NA	NA	NA	NA	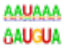	23.48/8.94<br>40.98/22.02	1e-53/0.001<br>1e-51/0.001
6	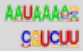	46.18/10.72<br>38.79/23.08	1e-289/0.001<br>1e-45/0.001	NA	NA	NA	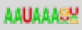	41.18/3.03	1e-24/0.001	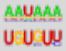	46.03/10.41<br>31.70/15.97	1e-1076/0.001<br>1e-200/0.001
7	NA	NA	NA	NA	NA	NA	NA	NA	NA	NA	NA	NA
9	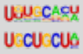	15.77/1.27<br>15.61/1.36	1e-71/0.001<br>1e-67/0.001	NA	NA	NA	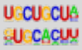	15.05/0.67<br>25.75/3.92	1e-44/0.001<br>1e-39/0.001	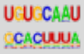	10.08/0.15<br>6.45/0.11	1e-36/0.001<br>1e-22/0.001
12	NA	NA	NA	NA	NA	NA	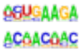	36.45/11.07<br>40.19/21.41	1e-42/0.001<br>1e-17/0.001	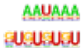	25.47/11.13<br>17.25/6.18	1e-26/0.001<br>1e-24/0.001
13	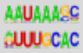	14.63/2.07<br>9.35/1.17	1e-19/0.001<br>1e-13/0.001	NA	NA	NA	NA	NA	NA	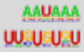	24.76/6.93<br>21.92/12.42	1e-65/0.001<br>1e-15/0.001
15	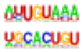	40.00/14.82<br>20.00/6.73	1e-32/0.001<br>1e-16/0.001	NA	NA	NA	NA	NA	NA	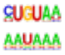	77.63/48.16<br>23.70/12.47	1e-204/0.001<br>1e-54/0.001
16	NA	NA	NA	NA	NA	NA	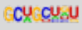	14.09/1.90	1e-11/0.045	NA	NA	NA
18	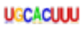	4.76/0.29	1e-17/0.001	NA	NA	NA	NA	NA	NA	NA	NA	NA

b

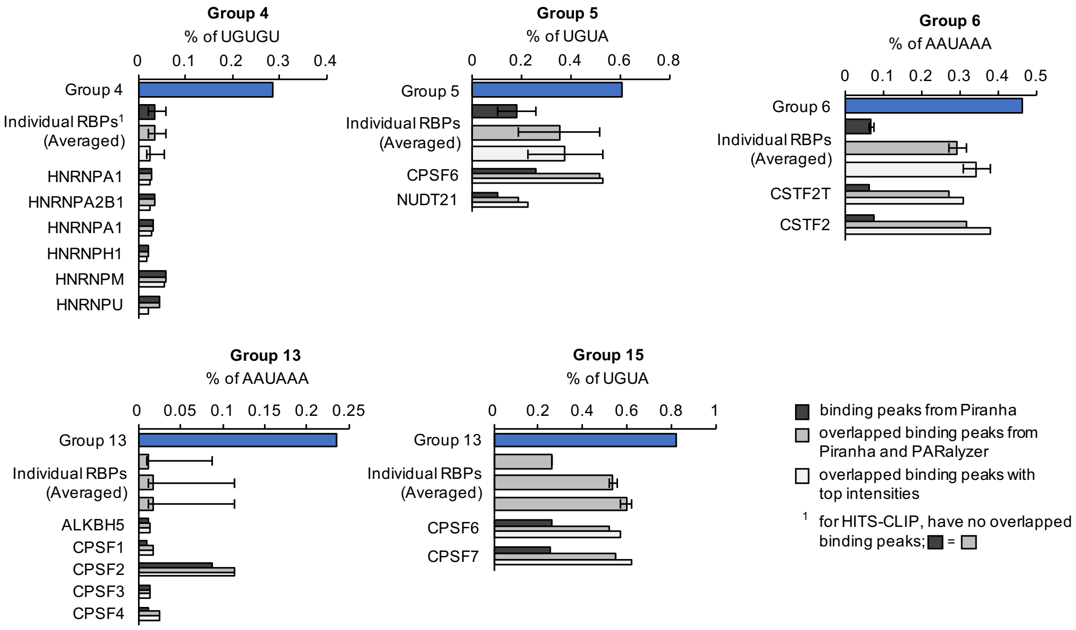

Figure S6 | The regulatory elements (motifs) for each RBP group in different regions and percentage of known motif in binding sites/peaks.

(a) In the main result, we predicted the enriched motifs for the whole transcripts. Here, group related RNA motifs were predicted by *de novo* motif finding for each genomic region, 5' UTR, 3' UTR, CDS and intron. The p-values of the motifs were calculated by binomial test against the randomly selected genome background. The FDR was calculated using HOMER (option "–fdr 1000", so the smallest number is 0.001). **(b)** The percentages of known motifs in the group related binding sites and individual RBPs' binding peaks. Usually, the related binding sites were less than the binding peaks called from individual RBP's data. Therefore, to make sure the comparison was fare, we used different thresholds to define the binding peaks identified by individual RBPs: 1) peaks called by Piranha only (p-value < 0.01); 2) peaks overlapped from two methods, Piranha and PARalyzer (default in the main text); 3) peaks with top binding intensities for individual RBPs were selected to make the total numbers of binding peaks/sites being compared were equal.

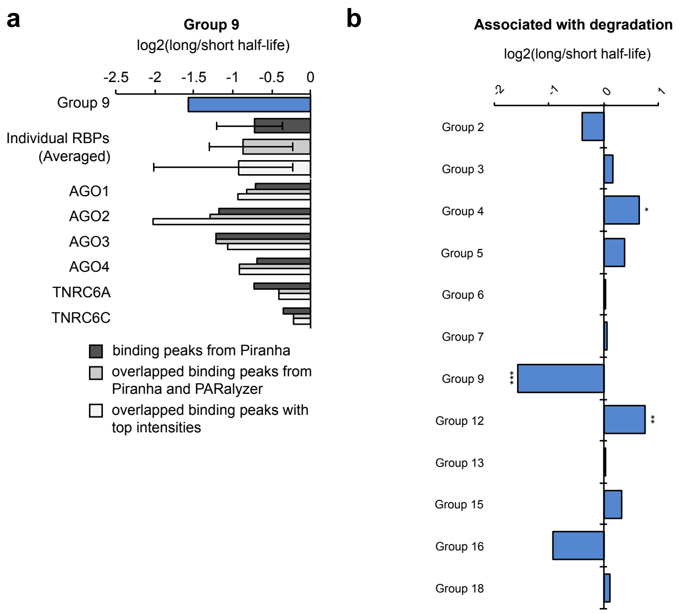

Figure S7 | The association between group related binding sites and degradation for each RBP group

(a) In Figure 5B, we calculated the log2 ratio of the fraction of long half-life genes (>= 80% quantile of half-lives) to the fraction of short half-life genes (<=20% quantile of half-lives) containing group related binding sites or individual RBP's binding peaks. Usually, the group related binding sites were less than the binding peaks called from individual RBP's data. Therefore, to make sure the comparison was fare, we used different thresholds to define the binding peaks identified by individual RBPs: 1) peaks called by Piranha only (p-value < 0.01); 2) peaks overlapped from two methods, Piranha and PARalyzer (default in the main text); 3) peaks with top binding intensities for individual RBPs were selected to make the total numbers of binding peaks/sites being compared were equal.

(b) We show the enrichment for the binding sites of every RBP group. Only Group 9 was found to be significantly correlated with RNA degradation. This was consistent with these RBPs' known function: no other RBP groups in our data set were reported to be related to RNA degradation (Figure 4B). (*, p-value<=0.01; **, p-value<0.005; ***, p-value<0.001, Fisher exact test).

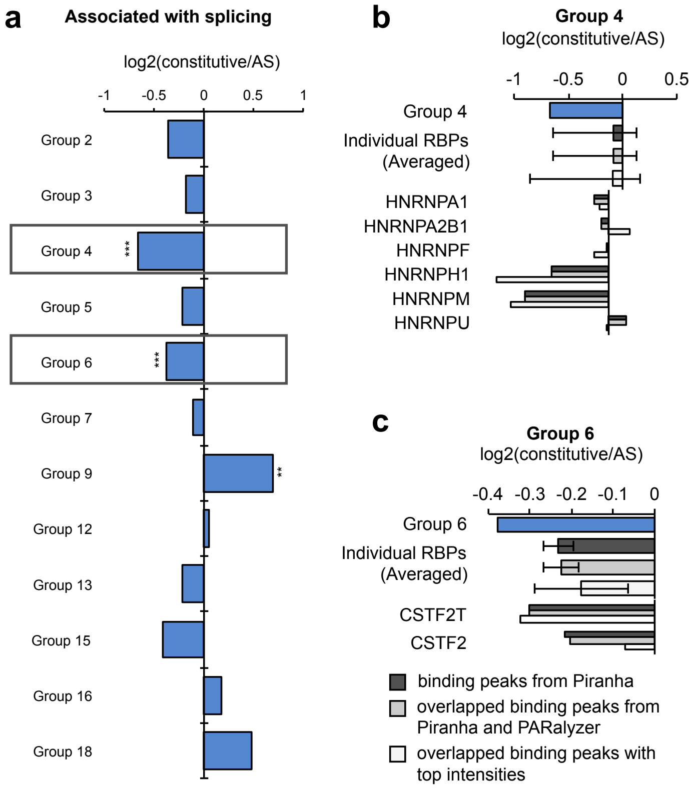

Figure S8 | The association between group related binding sites and alternative splicing for each RBP group

(a) The group related binding sites were used to infer its function by calculating the log2 ratio of the fraction of constitutive exons (PSI score >= 0.8) to the fraction of cassette exons (PSI score <= 0.2) containing group related binding sites within 2 kb upstream and downstream of exon. The significance depends on the size of test population (**, *p*-value<0.005, ***, *p*-value<0.001, Fisher exact test).

(b) We calculating the log2 ratio of the fraction of constitutive exons to the fraction of cassette exons for group related binding sites and individual RBP binding peaks. Usually, the group related binding sites were less than the binding peaks called from individual RBP's data. Therefore, to make sure the comparison was fare, we used different thresholds to define the binding peaks identified by individual RBPs: 1) peaks called by Piranha only (*p*-value < 0.01); 2) peaks overlapped from two methods, Piranha and PARalyzer (default in the main text); 3) peaks with top binding intensities for individual RBPs were selected to make the total numbers of binding peaks/sites being compared were equal.

(c) The same as **(b)** for group 6.

a

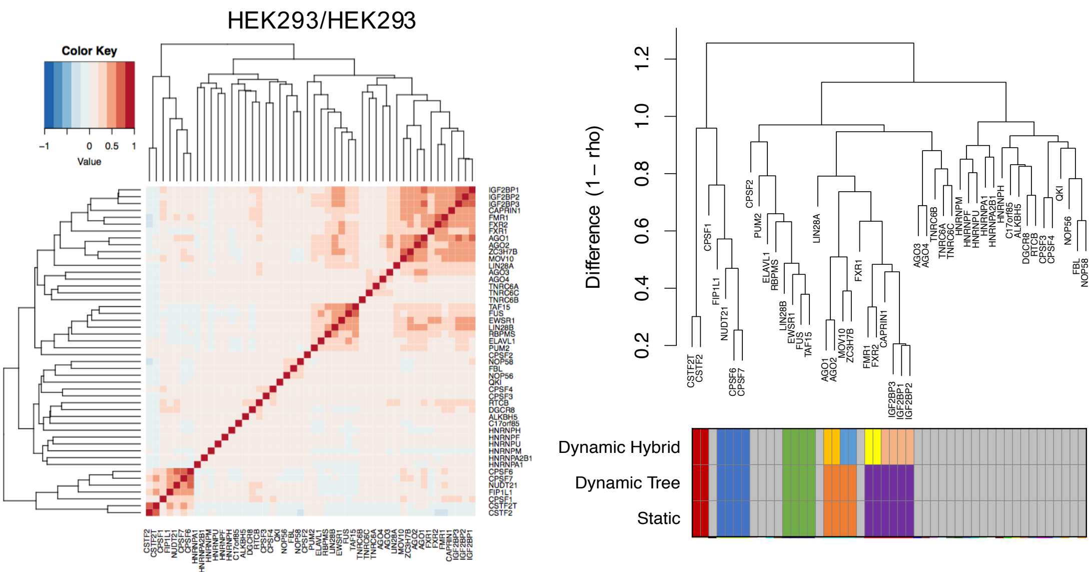

Cluster	Components	Physical interactions (*p*-value)	Overlap with group # (NMF method)
1	CSTF2T, CSTF2	0.53735	6
2	FIP1L1, NUDT21, CPSF6, CPSF7	**0.00925**	5, 14, 15
3	ELAVL1, RBPMS, EWSR1, FUS, LIN28B, TAF15	0.08295	2, 3
4	AGO1, AGO2, MOV10, ZC3H7B	**0.04480**	8, 9, 17
5	FMR1, FXR2, CAPRIN1, IGF2BP1, IGF2BP2, IGF2BP3	0.49860	1, 10, 18

b

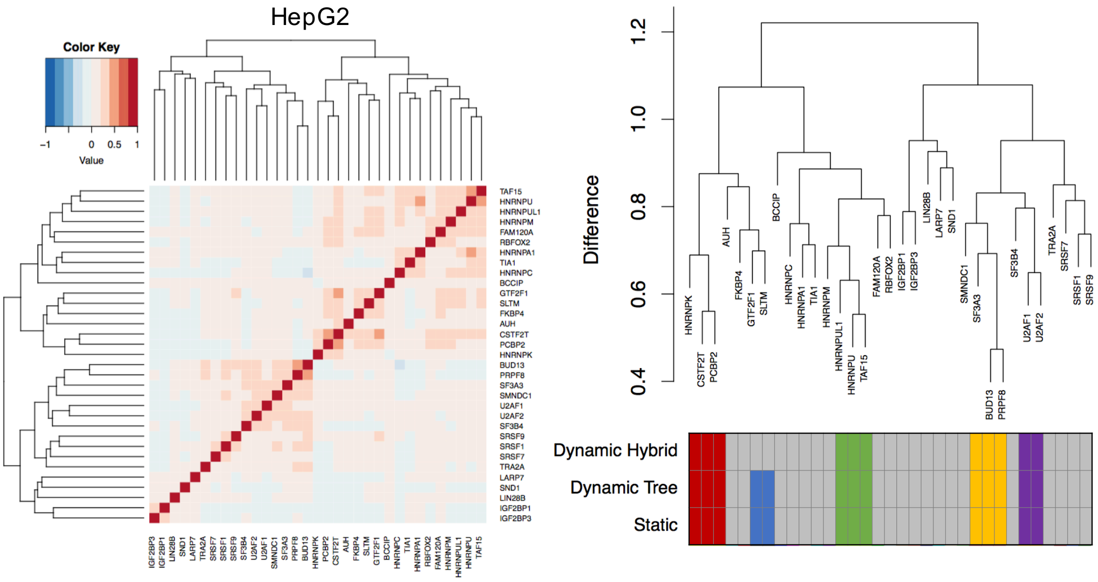

Cluster	Components	Physical interactions (*p*-value)	Overlap with group # (NMF method)
1	HNRNPK, CSTF2T, PCBP2	0.16325	3
2	GTF2F1, SLTM	0.50000	--
3	HNRNPUL1, HNRNPU, TAF15	0.64005	2
4	SF3A3, BUD13, PRPF8	0.16325	11
5	U2AF1, U2AF2	0.50000	10

c

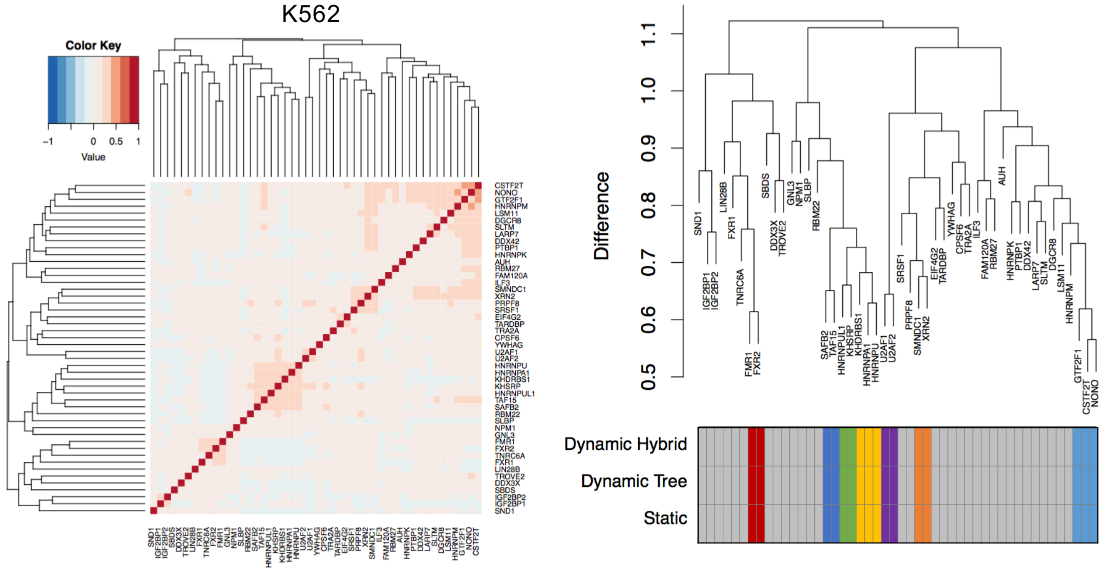

Cluster	Components	Physical interactions (p-value)	Overlap with group # (NMF method)
1	FMR1,FXR2	**0.03735**	16
2	SAFB2,TAF15	0.53735	--
3	HNRNPUL1,KHSRP	0.53735	--
4	KHDRBS1,HNRNPA1,HNRNPU	**0.01560**	2
5	U2AF1,U2AF2	**0.03735**	6
6	SMNDC1,XRN2	0.53735	--
7	GTF2F1,CSTF2T,NONO	0.59985	10

Figure S9 | Hierarchical clustering of Spearman correlation coefficients between any two RBPs

We show Figure 3A with more details here (**a**) and added the analyses for the data in two other cell lines (**b, c**). The co-binding pattern of multiple RBPs, we calculated the pairwise similarity (Spearman correlation coefficients, *rho,* indicates the similarity) between RBPs using occupancy profile matrix in HEK293/HEK293T **(a)**, HepG2 **(b)** and K562 **(c)** cell lines. We choose three methods to define RBP groups from the hierarchical cluster tree. The "static" method use a constant height cut-off, $1 - \text{rho}$, to define clusters. Most of the RBPs are still singleton and cannot be clustered together even when we used a very low similarity cutoff (rho=0.4 for HEK293/HEK293T dataset; rho=0.3 for HepG2 and K562 datasets). We also used two other methods, Dynamic Hybrid and Dynamic Tree to cluster the RBPs (minimum cluster size: 2). Comparing with NMF methods, the conventional Spearmen correlation clustering identify less clusters with little physical interaction support.

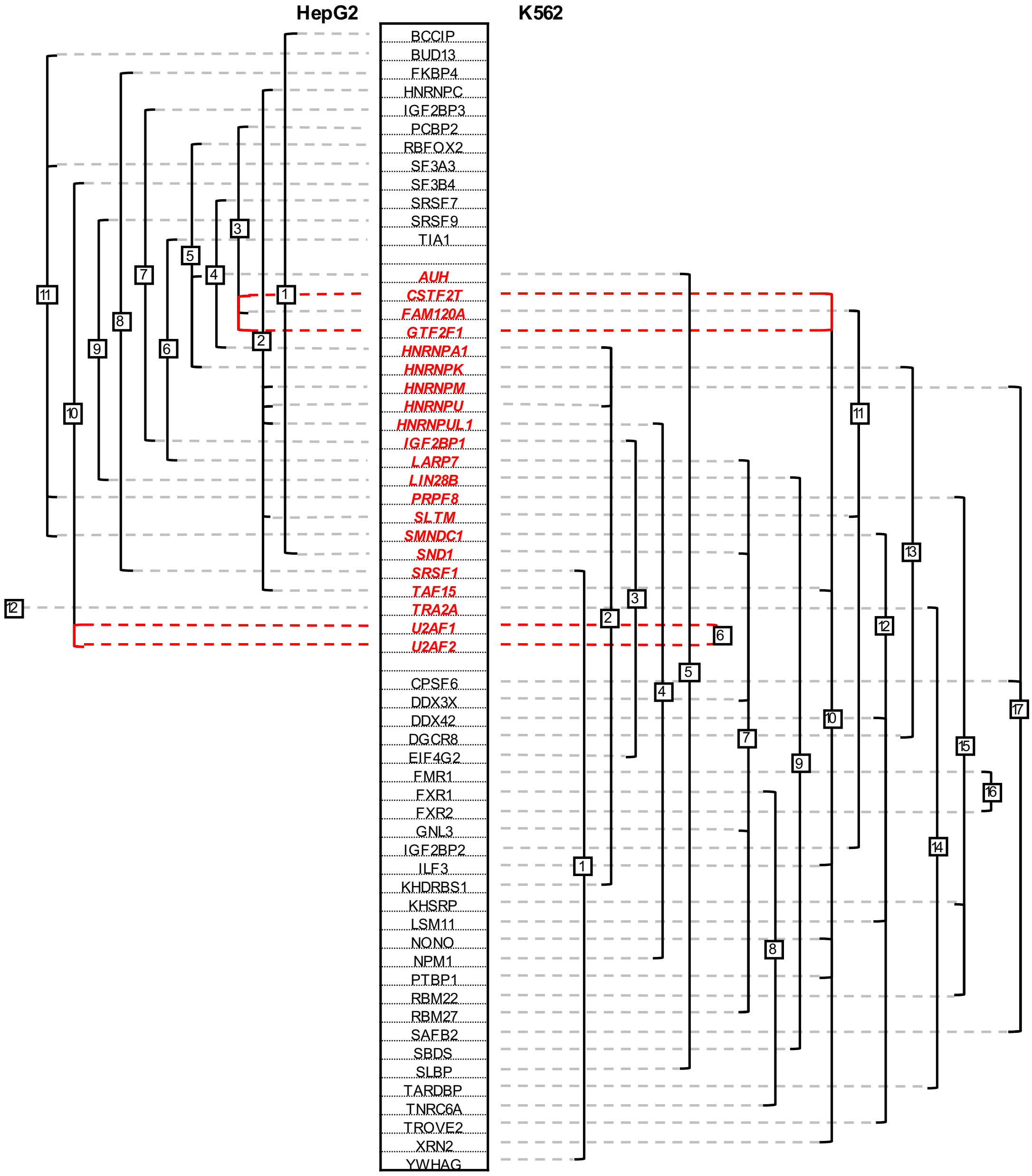

Figure S10 | The comparison of RBP groups between HepG2 and K562 datasets.

The RBPs shared by the two cell lines are labeled in red. HepG2-only and K562-only RBPs are listed at the top and bottom, respectively. Group numbers are labeled in the square boxes. Some RBPs, such as CSTF2T-GTF2F1 and U2AF1-U2AF2, are clustered together in the same groups (red lines) in both cell lines.

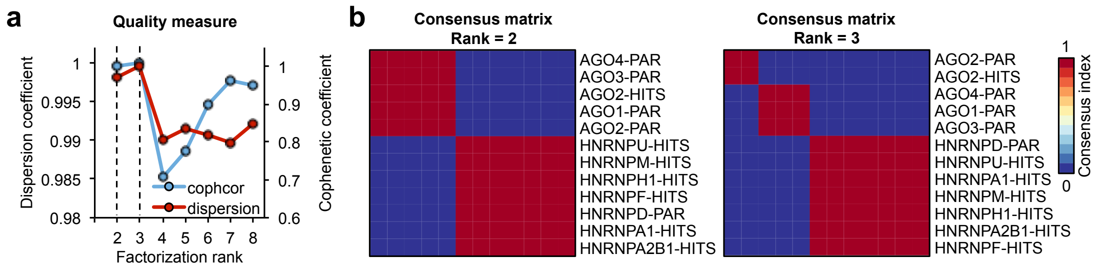

Figure S11 | RBPs in the same family were clearly clustered together without influenced by technical bias

To check whether technical batch effect may contribute to the clustering, we generated a new dataset using AGO1-4 from PAR-CLIP, AGO2 from HITS-CLIP, HNRNPD from PAR-CLIP and HNRNPA1/A2B1/F/H1/M/U from HITS-CLIP in the HEK293/HEK293T cell line. **(a)** According to two quality measures, cophenetic and dispersion coefficient, we performed the NMF at rank 2 and 3, respectively. **(b)** As the consensus matrix shown, RBPs in the same family were clearly clustered together without influenced by technical bias.

a

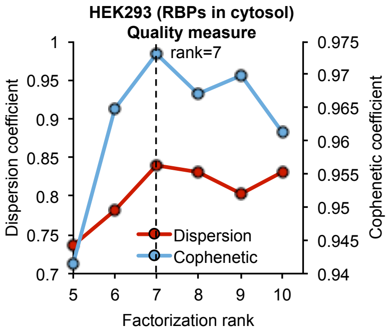

b

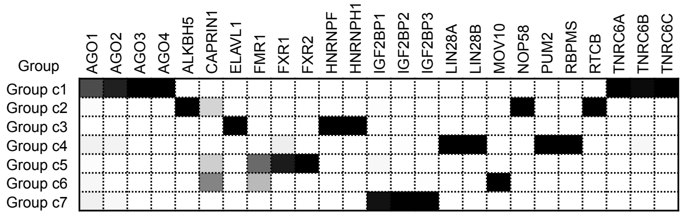

Group	Components	Size	Num. of supported physical interactions	*p*-value
Group c1	AGO1,AGO2,AGO3,AGO4,TNRC6A,TNRC6B,TNRC6C	7	16	**0.0000**
Group c2	ALKBH5,NOP58,RTCB	3	0	0.6077
Group c3	ELAVL1,HNRNPF,HNRNPH1	3	1	0.1178
Group c4	LIN28A,LIN28B,PUM2,RBPMS	4	0	0.6885
Group c5	FMR1,FXR1,FXR2	3	3	**0.0018**
Group c6	CAPRIN1,FMR1,MOV10	3	0	0.6077
Group c7	IGF2BP1,IGF2BP2,IGF2BP3	3	0	0.6077

Figure S12 | NMF analysis for cytosolic RBPs

We selected 25 cytosolic RBPs (confidence score = 5) and used polyA RNA-seq (GSE68671) for the normalization. Then, we performed NMF. **(a)** Criteria for estimating rank R in NMF. The cophenetic correlation coefficient (CPCC) and dispersion coefficient (DC) quantitatively measure the stability of clustering associated with each rank R, based on a consensus matrix. **(b)** Heatmap shows the weights of each RBP in each group, which were derived from the coefficient matrix. The values of each column are scaled (0 – 1). 7 RBP groups were inferred from 25 cytosolic RBPs, supported by known physical interactions derived from GeneMANIA database. The p-value was estimated from 10,000 random RBP sets for each group.

a b

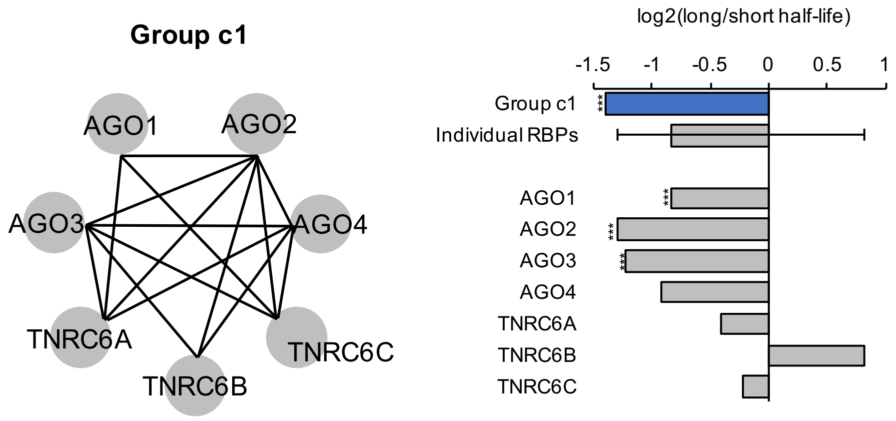

Figure S13 | The association between group related binding sites and RNA degradation for group c1

(a) Known physical interactions between RBPs in group c1.

(b) We calculated the fractions (log2 ratios) having binding sites co-bound by group c1 (or binding peaks called from individual RBP's CLIP-seq data) for long half-life genes and short half-life genes. RNA binding sites co-bound by RBP group c1 are significantly enriched in short half-life genes (Fisher's exact test, p-value = 4.64E-05) (***, p-value<0.001). The enrichment of group c1 is also better than the average of individual RBPs.

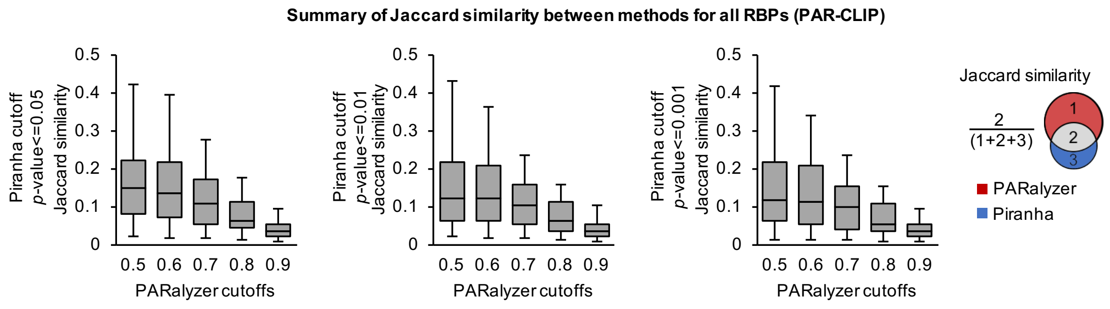

Figure S14 | Jaccard similarity between peak calling methods for three cell lines

We set different cutoffs to Piranha (*p*-value smaller than 0.05, 0.01, 0.001, respectively) and PARalyzer (the ModeScore from 0.5 to 0.9, which represents the "strength" of the signal from that cluster) for peak calling. The boxplot shows the summary of Jaccard similarity between peak calling methods with different cutoffs for all RBPs (PAR-CLIP).

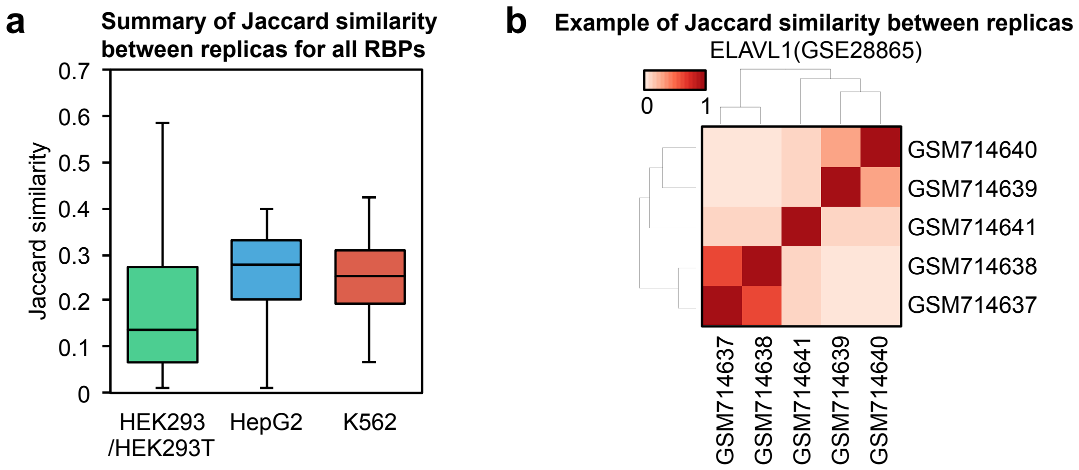

Figure S15 | Jaccard similarity between replicas for three cell lines

(a) The boxplot shows the summary of Jaccard similarity between replicas using the same peak calling methods (Piranha for HEK293 cell line, CLIPper for HepG2 and K562 cell line) in three cell lines.

(b) We use ELAVL1 (GSE28865) as an example to show the discrepancy of binding sites between biological replicas. Heatmap shows the Jaccard similarity between pairwise samples from the same GSE ID. The binding sites are identified using Piranha (*p*-value<0.01).

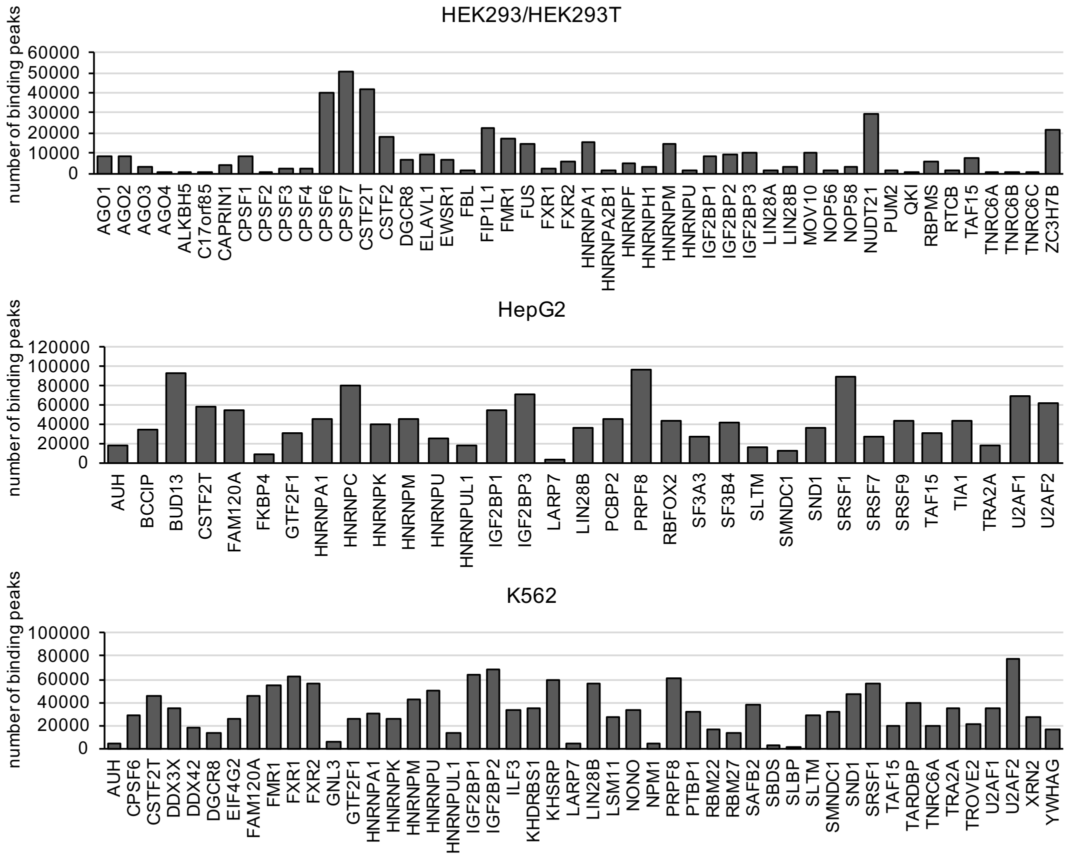

Figure S16 | The number of binding peaks selected for CLIP-seq data in three cell lines

a

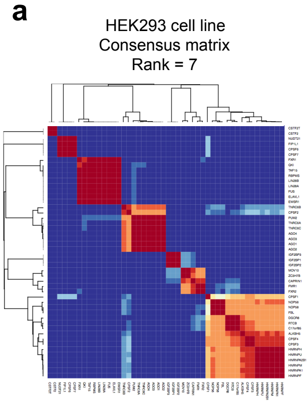

b

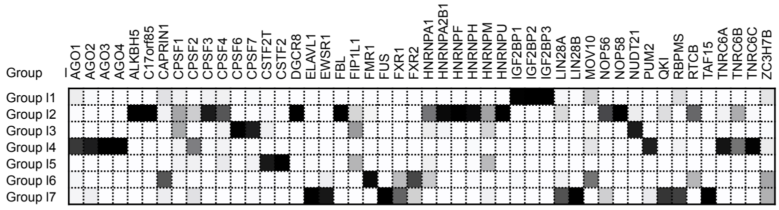

Group	Components	Size	Num. of supported physical interactions	*p*-value
Group I1	IGF2BP1, IGF2BP2, IGF2BP3, MOV10	4	1	0.2165
Group I2	ALKBH5, C17orf85, CPSF1, CPSF2, CPSF3, CPSF4, DGCR8, FBL, HNRNPA1, HNRNPA2B1, HNRNPF, HNRNPH, HNRNPM, HNRNPU, NOP56, NOP58, RTCB, TNRC6B	18	21	**0.0210**
Group I3	CPSF1, CPSF6, CPSF7, FIP1L1, NUDT21	5	4	**0.0080**
Group I4	AGO1, AGO2, AGO3, AGO4, CPSF2, PUM2, TNRC6A, TNRC6B, TNRC6C	9	16	**0.0010**
Group I5	CSTF2T, CSTF2, FIP1L1, HNRNPM	4	0	0.6820
Group I6	CAPRIN1, FMR1, FXR1, FXR2, HNRNPA1, MOV10, RTCB, ZC3H7B	8	4	0.1385
Group I7	ELAVL1, EWSR1, FUS, FXR1, FXR2, LIN28A, LIN28B, NOP56, QKI, RBPMS, TAF15, ZC3H7B	12	7	0.2435

Figure S17 | NMF analysis on low rank.

We showed the result derived from NMF on a low rank, 7. **(a)** The consensus matrix on rank 7. **(b)** Heatmap shows the weights of each RBP in each group, which were derived from the coefficient matrix. The values of each column are scaled (0 – 1). 7 RBP groups were inferred from 48 RBPs in HEK293 dataset. The supporting information of known physical interactions was derived from GeneMANIA database; the *p* value was estimated from 10,000 random RBP sets for each group.

Supplementary Tables

Table S1 | Human RNA-binding proteins collected for HEK293/HEK293T cell lines

Factor	Cell line	Assay	Data accession	Putative RNA target	Localization* nucleus/cytosol	Functional annotation	Reference
AGO1	HEK293	PAR-CLIP	GSE43573 ; GSM1065667, GSM1065668, GSM1065669	miRNA/mRNA	5 / 5	miRNA binding; Poly(A) RNA binding; protein binding	[2]
AGO2	HEK293	PAR-CLIP	GSE28865; GSM714644, GSM714645	miRNA/mRNA	5 / 4	RNA 7-methylguanosine cap binding; mRNA binding; miRNA binding; poly(A) RNA binding; siRNA binding	[3]
AGO3	HEK293	PAR-CLIP	GSE21578; GSM545214	miRNA/mRNA	5 / 5	miRNA binding; poly(A) RNA binding	[4]
AGO4	HEK293	PAR-CLIP	GSE21578; GSM545215	miRNA/mRNA	5 / 5	miRNA binding; protein binding	[4]
ALKBH5	HEK293	PAR-CLIP	GSE38201; GSM936506	mRNA	5 / 5	poly(A) RNA binding; m6A mRNA demethylation	[5]
C17orf85	HEK293	PAR-CLIP	GSE38201; GSM936507	unknown	5 / 2	Nucleotide binding; poly(A) RNA binding	[5]
CAPRIN1	HEK293	PAR-CLIP	GSE38201; GSM936509	mRNA	3 / 5	poly(A) RNA binding	[5]
CPSF1	HEK293	PAR-CLIP	GSE37401; GSM917672, GSM917673	mRNA	5 / 2	mRNA 3' UTR binding; poly(A) RNA binding; protein binding	[6]
CPSF2	HEK293	PAR-CLIP	GSE37401; GSM917670, GSM917671	mRNA	5 / 1	mRNA 3' UTR binding; poly(A) RNA binding; protein binding	[6]

CPSF3	HEK293	PAR-CLIP	GSE37401; GSM917668, GSM917669	mRNA	5 / 2	mRNA 3’ UTR binding; poly(A) RNA binding; protein binding	[6]
CPSF4	HEK293	PAR-CLIP	GSE37401; GSM917666, GSM917667	mRNA	5 / 1	mRNA 3’ UTR binding; poly(A) RNA binding; protein binding	[6]
CPSF6	HEK293	PAR-CLIP	GSE37401; GSM917664, GSM917665	mRNA	5 / 2	mRNA 3’ UTR binding; poly(A) RNA binding; pre-mRNA splicing; protein binding	[6]
CPSF7	HEK293	PAR-CLIP	GSE37401; GSM917663	mRNA	5 / 3	mRNA 3’ UTR binding; poly(A) RNA binding; pre-mRNA splicing; protein binding	[6]
CSTF2	HEK293	PAR-CLIP	GSE37401; GSM917676	mRNA	5 / 1	poly(A) RNA binding; mRNA splicing	[6]
CSTF2T	HEK293	PAR-CLIP	GSE37401; GSM917677, GSM917678	mRNA	5 / 2	poly(A) RNA binding; mRNA processing	[6]
DGCR8	HEK293T	HITS-CLIP	GSE39086; GSM955510, GSM955511, GSM955512, GSM955513	miRNA	5 / 3	Double-stranded RNA binding; protein binding	[7]
ELAVL1	HEK293	PAR-CLIP	GSE28865; GSM714637, GSM714638	mRNA	5 / 5	mRNA 3'-UTR binding; poly(A) RNA binding	[3]
EWSR1	HEK293	PAR-CLIP	SRX029343, SRX029344	mRNA	5 / 2	poly(A) RNA binding; protein binding	[8]

FBL	HEK293	PAR-CLIP	GSE43666; GSM1067864, GSM1067865	snoRNA	5 / 2	poly(A) RNA binding	[9]
FIP1L1	HEK293	PAR-CLIP	GSE37401; GSM917674, GSM917675	mRNA	5 / 2	poly(A) RNA binding	[6]
FMR1	HEK293	PAR-CLIP	GSE39682; GSM977615, GSM977616	mRNA	5 / 5	mRNA binding; poly(A) RNA binding; protein binding; translation repressor	[10]
FUS	HEK293	PAR-CLIP	SRX029328, SRX029329	mRNA	5 / 2	poly(A) RNA binding; protein binding	[8]
FXR1	HEK293	PAR-CLIP	GSE39682; GSM977619	mRNA	5 / 5	G-quadruplex RNA binding; mRNA 3' UTR binding; poly(A) RNA binding; protein binding	[10]
FXR2	HEK293	PAR-CLIP	GSE39682; GSM977620	mRNA	5 / 5	poly(A) RNA binding; protein binding	[10]
HNRNPA1	HEK293T	HITS-CLIP	GSE34996; GSM859978, GSM859979, GSM859980, GSM859981	mRNA	5 / 1	poly(A) RNA binding; mRNA splicing	[11]
HNRNPA2B1	HEK293T	HITS-CLIP	GSE34996; GSM859982	mRNA	5 / 1	poly(A) RNA binding; mRNA splicing	[11]
HNRNPF	HEK293T	HITS-CLIP	GSE34996; GSM859983, GSM859984, GSM859985, GSM859986	mRNA	5 / 5	poly(A) RNA binding; mRNA splicing	[11]

HNRNPH1	HEK293T	HITS-CLIP	GSE23694; GSM581051, GSM581052	mRNA	5 / 5	poly(A) RNA binding; mRNA splicing	[12]
HNRNPM	HEK293T	HITS-CLIP	GSE34996; GSM859987, GSM859988	mRNA	5 / 2	poly(A) RNA binding; mRNA splicing	[11]
HNRNPU	HEK293T	HITS-CLIP	GSE34996; GSM859989, GSM859990, GSM859991, GSM859992	mRNA	5 / 2	poly(A) RNA binding; mRNA splicing	[11]
IGF2BP1	HEK293	PAR-CLIP	GSE21578; GSM545206, GSM545207	mRNA	5 / 5	mRNA 3' UTR binding; mRNA 5' UTR binding; poly(A) RNA binding; protein binding; translation regulator activity	[4]
IGF2BP2	HEK293	PAR-CLIP	GSE21578; GSM545208	mRNA	5 / 5	mRNA 3' UTR binding; mRNA 5' UTR binding; poly(A) RNA binding; protein binding; translation regulator activity	[4]
IGF2BP3	HEK293	PAR-CLIP	GSE21578; GSM545209	mRNA	5 / 5	mRNA 3' UTR binding; mRNA 5' UTR binding; poly(A) RNA binding; protein binding; translation regulator activity	[4]
LIN28A	HEK293	PAR-CLIP	GSE44616; GSM1087848	mRNA/miRNA/snoRNA	5 / 5	mRNA binding; miRNA binding; protein binding;	[13]

						translation initiation factor binding; translational enhancer	
LIN28B	HEK293	PAR-CLIP	GSE44616; GSM1087849, GSM1087850, GSM1087851	mRNA/miRNA/snoRNA	5 / 5	RNA binding; poly(A) RNA binding; protein binding; miRNA precursor let-7 binding	[13]
MOV10	HEK293	PAR-CLIP	GSE37524; GSM921128	mRNA	2 / 5	ATP binding; helicase activity; poly(A) RNA binding; protein binding	[14]
NOP56	HEK293	PAR-CLIP	GSE43666; GSM1067863	snoRNA	5 / 2	poly(A) RNA binding; snoRNA binding; protein binding	[9]
NOP58	HEK293	PAR-CLIP	GSE43666; GSM1067861, GSM1067862	snoRNA	5 / 5	poly(A) RNA binding; snoRNA binding; protein binding	[9]
NUDT21	HEK293	PAR-CLIP	GSE37401; GSM917661, GSM917662	mRNA	5 / 1	AU-rich element binding; poly(A) RNA binding; mRNA binding; protein binding (CPSF6 CPSF7)	[6]
PUM2	HEK293	PAR-CLIP	GSE21578; GSM545210	mRNA	5 / 5	mRNA 3' UTR binding; poly(A) RNA binding;	[4]
QKI	HEK293	PAR-CLIP	GSE21578; GSM545211	mRNA	5 / 2	poly(A) RNA binding; mRNA splicing	[4]
RBPMS	HEK293	PAR-CLIP	SRX484627, SRX484628	mRNA	5 / 5	poly(A) RNA binding; mRNA processing	[15]
RTCB	HEK293	PAR-CLIP	GSE38201; GSM936508	tRNA	5 / 5	poly(A) RNA binding	[5]
TAF15	HEK293	PAR-CLIP	SRX029345, SRX029346	mRNA	5 / 2	poly(A) RNA binding; protein binding	[8]
TNRC6A	HEK293	PAR-CLIP	GSE21578; GSM545218	mRNA	5 / 5	poly(A) RNA binding; protein binding	[4]

TNRC6B	HEK293	PAR-CLIP	GSE21578; GSM545219	mRNA	3 / 5	poly(A) RNA binding; protein binding	[4]
TNRC6C	HEK293	PAR-CLIP	GSE21578; GSM545220	mRNA	3 / 5	RNA binding; protein binding	[4]
ZC3H7B	HEK293	PAR-CLIP	GSE38201; GSM936510	unknown	4 / 3	poly(A) RNA binding; protein binding	[5]

* Sub-cellular localization information according to UniProtKB (http://www.uniprot.org/help/uniprotkb) and COMPARTMENTS Subcellular localization database (http://compartments.jensenlab.org/Search). The localization information in the databases was derived from database annotations, automatic text mining of the biomedical literature, and sequence-based predictions. Confidence score scales from 1 to 5, e.g. 1 for low confidence and 5 for high confidence.

Table S2 | The *p*-value of supporting evidence for each RBP group in HEK293/HEK293T cell lines

Group	Co-expression	Shared protein domains	Genetic interactions	Physical interactions	Pathway	Predicted	Co-localization	Components
1	0.5	0.5	0.5	0.5	0.5	0.5	0.5	FMR1
2	0.954	0.53305	0.4126	0.3713	0.91225	0.8678	0.86395	AGO4, CPSF2, ELAVL1, LIN28A, PUM2, QKI, RBPMS, TNRC6A, TNRC6B, TNRC6C
3	0.35995	**0.03565**	0.61065	**0.0448**	0.6485	**0.00915**	0.59345	EWSR1, FUS, LIN28B, TAF15
4	**0.0005**	0.31885	0.72215	**0.0000**	**1.00E-04**	0.6894	**0.00015**	HNRNPA1, HNRNPA2B1, HNRNPF, HNRNPH1, HNRNPM, HNRNPU
5	0.2122	0.552	0.5221	0.03735	0.5355	0.01465	0.51795	CPSF6, NUDT21
6	0.2122	**0.052**	0.5221	0.53735	0.5355	0.51465	0.51795	CSTF2T, CSTF2
7	0.7122	0.552	**0.0221**	0.53735	0.5355	0.51465	0.51795	C17orf85, RTCB
8	0.5	0.5	0.5	0.5	0.5	0.5	0.5	ZC3H7B
9	0.2558	**0.01045**	0.2934	**0.0000**	0.7664	**0.0002**	0.2413	AGO1, AGO2, AGO3, AGO4, TNRC6A, TNRC6C
10	0.5	0.5	0.5	0.5	0.5	0.5	0.5	CAPRIN1
11	0.5	0.5	0.5	0.5	0.5	0.5	0.5	DGCR8

12	0.0555	0.15335	0.5571	**0.0018**	0.58855	**0.0005**	0.0521	FBL, NOP56, NOP58
13	0.5203	0.43495	0.6625	**0.0092**	0.0677	**0.0000**	0.1679	ALKBH5, CPSF1, CPSF2, CPSF3, CPSF4
14	0.5	0.5	0.5	0.5	0.5	0.5	0.5	FIP1L1
15	0.2122	0.052	0.5221	**0.03735**	0.5355	0.51465	0.51795	CPSF6, CPSF7
16	0.5676	0.15335	0.5571	**0.0119**	0.58855	**0.04735**	0.5485	EWSR1, FXR1, FXR2
17	0.5	0.5	0.5	0.5	0.5	0.5	0.5	MOV10
18	0.0555	**0.0081**	0.0603	0.60765	0.58855	0.5459	0.5485	IGF2BP1, IGF2BP2, IGF2BP3

The table is showing *p*-value (Supplementary Figure 5) of each evidence type for each group. The evidence of known association was derived from GeneMANIA database [1].

Table S3 | The *p*-value of supporting evidence for each RBP group in HepG2 cell line (ENCODE)

Group	Co-expression	Genetic Interactions	Shared protein domains	Co-localization	Pathway	Predicted	Physical Interactions	Components
1	0.7762	0.52045	0.6077	0.53535	0.60935	0.5175	0.5531	BCCIP, SND1
2	0.0542	0.70805	0.1559	0.2201	0.18655	0.67855	0.6239	HNRNPC, HNRNPM, HNRNPU, HNRNPUL1, SLTM, TAF15
3	0.92255	0.59855	0.8383	0.6676	0.4784	0.5881	0.7339	CSTF2T, FAM120A, GTF2F1, PCBP2
4	0.2762	0.52045	0.6077	0.53535	0.10935	0.5175	**0.0531**	HNRNPA1, SRSF7
5	0.378	0.554	0.7416	0.6003	0.73675	0.54485	0.16325	AUH, HNRNPK, RBFOX2
6	0.7762	0.52045	0.1077	0.53535	0.60935	0.5175	0.5531	LARP7, TIA1
7	0.2762	0.52045	0.1077	0.53535	0.60935	0.5175	0.5531	IGF2BP1, IGF2BP3
8	0.7762	0.52045	0.6077	0.53535	0.60935	0.5175	0.5531	FKBP4, SRSF1
9	0.7762	0.52045	0.6077	0.53535	0.60935	0.5175	0.5531	LIN28B, SRSF9
10	0.378	0.554	**0.0327**	0.6003	**0.0519**	**0.0468**	0.16325	SF3B4, U2AF1, U2AF2
11	**0.0472**	0.1108	0.8383	0.2077	0.4784	**0.0116**	0.1046	BUD13, PRPF8, SF3A3, SMNDC1
12	0.5	0.5	0.5	0.5	0.5	0.5	0.5	TRA2A

The table is showing *p*-value (Supplementary Figure 5) of each evidence type for each group. The evidence of known association was derived from GeneMANIA database [1].

Table S4 | The *p*-value of supporting evidence for each RBP group in K562 cell line (ENCODE)

Group	Co-expression	Shared protein domains	Genetic Interactions	Co-localization	Pathway	Predicted	Physical Interactions	Components
1	0.2605	0.56575	0.5156	0.519	0.5247	0.5198	**0.03735**	SRSF1, YWHAG
2	0.104	0.65965	0.54225	**0.00065**	0.07125	**0.00475**	**0.0156**	HNRNPA1, HNRNPU, KHDRBS1
3	0.7605	0.56575	0.5156	0.519	0.5247	0.5198	0.53735	EIF4G2, IGF2BP1
4	0.7605	0.56575	0.5156	0.519	0.5247	0.5198	0.53735	HNRNPUL1, NPM1
5	0.2605	0.56575	0.5156	0.519	0.5247	0.5198	0.53735	AUH, SLBP
6	0.7605	0.06575	0.5156	0.519	**0.0247**	**0.0198**	**0.03735**	U2AF1, U2AF2
7	0.84465	0.83425	0.62075	0.6463	0.6676	0.6544	0.32945	DDX3X, GNL3, LARP7, RBM27, SND1
8	0.7605	0.56575	0.5156	0.519	0.5247	0.5198	0.53735	FXR1, TNRC6A
9	0.7605	0.56575	0.5156	0.519	0.5247	0.5198	0.53735	LIN28B, SBDS
10	0.06865	0.11865	0.36105	0.2049	0.4591	0.45075	0.8903	CSTF2T, GTF2F1, ILF3, NONO, PTBP1, SLTM, TAF15, XRN2
11	0.7605	0.56575	0.5156	0.519	0.5247	0.5198	0.53735	FAM120A, IGF2BP2
12	0.5201	0.75355	0.57785	0.5946	0.61535	0.60285	0.6657	DDX42, LSM11, SMNDC1, TROVE2

13	0.7605	0.56575	0.5156	0.519	0.5247	0.5198	0.53735	DGCR8, HNRNPK
14	0.2605	0.06575	0.5156	0.519	0.5247	0.5198	0.53735	TARDBP, TRA2A
15	0.35795	0.65965	0.54225	0.55365	0.566	**0.05975**	0.59985	KHSRP, PRPF8, RBM22
16	0.7605	0.06575	0.5156	0.519	0.5247	**0.0198**	**0.03735**	FMR1, FXR2
17	0.35795	**0.03855**	0.54225	0.55365	0.566	0.55545	0.59985	CPSF6, HNRNPM, SAFB2

The table is showing *p*-value (Supplementary Figure 5) of each evidence type for each group. The evidence of known association was derived from GeneMANIA database [1].

Table S5 | Nucleotide content in the sequences for motif identification

We have compared the proportion of nucleotides (A, C, G and U) for motif finding between target (group related binding sites) and background sequences (used for de novo motif finding). For each group, the proportion of nucleotides has no significantly difference between target and background sequences by Student's t-test, even when we compared sequences in 3'-UTR, 5'-UTR, CDS and intron regions.

Group	Region	Target sequences (%)				Background sequences (%)				p-value
		A	C	G	U	A	C	G	U	
Group 1	total	0.28	0.21	0.22	0.29	0.27	0.20	0.22	0.30	0.99994
	3'-UTR	0.30	0.19	0.19	0.32	0.28	0.18	0.20	0.34	0.99999
	5'-UTR	0.23	0.26	0.27	0.24	0.21	0.25	0.28	0.26	0.99955
	CDS	0.26	0.23	0.24	0.27	0.27	0.23	0.25	0.26	0.99962
	Intron	0.30	0.19	0.19	0.32	0.29	0.18	0.21	0.33	0.99998
Group 2	total	0.31	0.18	0.18	0.33	0.28	0.19	0.17	0.37	1.00000
	3'-UTR	0.31	0.18	0.18	0.33	0.28	0.20	0.17	0.36	0.99996
	5'-UTR	0.27	0.22	0.23	0.28	0.26	0.22	0.22	0.30	0.99991
	CDS	0.26	0.23	0.24	0.27	0.27	0.25	0.22	0.26	0.99970
	Intron	0.33	0.16	0.17	0.34	0.28	0.17	0.16	0.39	1.00000
Group 3	total	0.32	0.17	0.17	0.34	0.31	0.16	0.17	0.36	0.99997
	3'-UTR	0.32	0.17	0.18	0.33	0.30	0.18	0.16	0.36	1.00000
	5'-UTR	0.24	0.25	0.25	0.25	0.24	0.25	0.24	0.27	0.99992
	CDS	0.29	0.20	0.21	0.30	0.31	0.21	0.19	0.29	0.99991
	Intron	0.33	0.16	0.17	0.34	0.31	0.16	0.17	0.36	0.99998
Group 4	total	0.31	0.18	0.18	0.33	0.29	0.16	0.19	0.36	1.00000

	3'-UTR	0.31	0.18	0.18	0.33	0.29	0.17	0.19	0.35	1.00000
	5'-UTR	0.17	0.31	0.33	0.18	0.13	0.33	0.34	0.19	0.99984
	CDS	0.28	0.21	0.22	0.29	0.31	0.19	0.24	0.26	1.00000
	Intron	0.32	0.17	0.18	0.33	0.29	0.16	0.19	0.36	1.00000
Group 5	total	0.30	0.19	0.19	0.32	0.28	0.18	0.20	0.34	0.99995
	3'-UTR	0.30	0.19	0.19	0.32	0.28	0.18	0.20	0.34	0.99995
	5'-UTR	0.22	0.28	0.28	0.23	0.19	0.27	0.29	0.25	0.99966
	CDS	0.24	0.25	0.26	0.25	0.24	0.25	0.27	0.25	0.99876
	Intron	0.31	0.18	0.18	0.33	0.28	0.17	0.19	0.35	0.99998
Group 6	total	0.32	0.17	0.17	0.34	0.29	0.16	0.17	0.37	0.99997
	3'-UTR	0.31	0.18	0.18	0.33	0.29	0.17	0.19	0.36	1.00000
	5'-UTR	0.30	0.19	0.20	0.31	0.29	0.18	0.21	0.33	0.99989
	CDS	0.30	0.19	0.19	0.32	0.28	0.18	0.20	0.34	0.99996
	Intron	0.32	0.17	0.17	0.34	0.29	0.16	0.17	0.38	0.99998
Group 7	total	0.29	0.20	0.21	0.30	0.27	0.19	0.21	0.32	0.99994
	3'-UTR	0.30	0.19	0.20	0.31	0.28	0.19	0.20	0.34	0.99997
	5'-UTR	0.33	0.16	0.17	0.34	0.35	0.16	0.16	0.32	0.99996
	CDS	0.26	0.23	0.24	0.27	0.27	0.23	0.24	0.26	0.99975
	Intron	0.30	0.19	0.19	0.32	0.27	0.18	0.20	0.35	0.99991
Group 8	total	0.33	0.16	0.17	0.34	0.31	0.16	0.17	0.37	0.99995
	3'-UTR	0.33	0.16	0.17	0.34	0.31	0.16	0.17	0.36	1.00000
	5'-UTR	0.31	0.18	0.19	0.32	0.27	0.19	0.19	0.35	0.99996
	CDS	0.30	0.19	0.19	0.32	0.31	0.19	0.19	0.31	0.99997
	Intron	0.33	0.16	0.16	0.35	0.31	0.15	0.17	0.38	0.99998
Group 9	total	0.30	0.19	0.20	0.31	0.27	0.20	0.20	0.33	0.99998

	3'-UTR	0.31	0.19	0.19	0.32	0.28	0.19	0.19	0.35	0.99997
	5'-UTR	0.21	0.28	0.29	0.22	0.17	0.30	0.29	0.24	0.99985
	CDS	0.27	0.22	0.22	0.28	0.27	0.22	0.22	0.28	0.99998
	Intron	0.30	0.19	0.19	0.32	0.28	0.19	0.19	0.34	0.99993
	total	0.29	0.20	0.21	0.30	0.28	0.20	0.21	0.32	0.99992
	3'-UTR	0.30	0.19	0.20	0.31	0.27	0.19	0.20	0.34	0.99991
Group 10	5'-UTR	0.26	0.23	0.24	0.27	0.25	0.21	0.27	0.28	0.99978
	CDS	0.26	0.23	0.24	0.27	0.27	0.23	0.23	0.27	0.99964
	Intron	0.30	0.19	0.19	0.32	0.29	0.19	0.19	0.34	1.00000
	total	0.28	0.21	0.21	0.30	0.26	0.20	0.22	0.32	0.99995
	3'-UTR	0.29	0.20	0.21	0.30	0.27	0.20	0.20	0.33	0.99999
Group 11	5'-UTR	0.21	0.28	0.29	0.22	0.20	0.27	0.30	0.23	0.99978
	CDS	0.25	0.24	0.24	0.27	0.26	0.23	0.25	0.26	1.00000
	Intron	0.30	0.19	0.20	0.31	0.26	0.19	0.20	0.35	0.99998
	total	0.30	0.19	0.19	0.32	0.30	0.19	0.19	0.32	0.99998
	3'-UTR	0.30	0.19	0.20	0.31	0.28	0.21	0.18	0.32	0.99991
Group 12	5'-UTR	0.26	0.23	0.24	0.27	0.24	0.24	0.23	0.28	0.99960
	CDS	0.29	0.21	0.21	0.30	0.32	0.20	0.22	0.26	1.00000
	Intron	0.32	0.17	0.18	0.33	0.30	0.18	0.17	0.35	0.99998
	total	0.30	0.19	0.19	0.32	0.27	0.19	0.19	0.35	0.99998
	3'-UTR	0.30	0.20	0.20	0.31	0.26	0.20	0.19	0.34	0.99997
Group 13	5'-UTR	0.21	0.28	0.29	0.22	0.20	0.28	0.29	0.23	0.99972
	CDS	0.26	0.23	0.24	0.27	0.26	0.24	0.23	0.27	0.99984
	Intron	0.31	0.18	0.19	0.32	0.27	0.18	0.18	0.36	0.99996
Group 14	total	0.31	0.18	0.18	0.33	0.28	0.17	0.18	0.36	0.99999

	3'-UTR	0.31	0.18	0.19	0.32	0.28	0.18	0.19	0.35	1.00000
	5'-UTR	0.21	0.28	0.29	0.22	0.18	0.27	0.30	0.25	0.99979
	CDS	0.23	0.26	0.27	0.24	0.22	0.27	0.26	0.25	0.99953
	Intron	0.32	0.17	0.18	0.33	0.29	0.17	0.18	0.37	0.99999
Group 15	total	0.33	0.16	0.16	0.35	0.30	0.15	0.17	0.38	1.00000
	3'-UTR	0.33	0.16	0.17	0.34	0.30	0.15	0.18	0.36	0.99999
	5'-UTR	0.27	0.22	0.23	0.28	0.25	0.24	0.22	0.29	0.99992
	CDS	0.28	0.21	0.21	0.30	0.28	0.20	0.22	0.30	0.99988
	Intron	0.33	0.16	0.16	0.35	0.31	0.15	0.17	0.38	1.00000
Group 16	total	0.30	0.19	0.20	0.31	0.29	0.20	0.19	0.32	0.99986
	3'-UTR	0.31	0.18	0.19	0.32	0.29	0.19	0.17	0.35	0.99996
	5'-UTR	0.28	0.21	0.22	0.29	0.25	0.20	0.23	0.32	0.99988
	CDS	0.28	0.22	0.22	0.29	0.29	0.22	0.21	0.28	0.99995
	Intron	0.31	0.18	0.18	0.33	0.30	0.17	0.18	0.35	1.00000
Group 17	total	0.31	0.18	0.18	0.33	0.29	0.18	0.18	0.35	0.99994
	3'-UTR	0.31	0.18	0.19	0.32	0.29	0.18	0.18	0.35	1.00000
	CDS	0.28	0.21	0.22	0.29	0.27	0.24	0.20	0.30	0.99986
	Intron	0.32	0.17	0.18	0.33	0.30	0.17	0.18	0.36	0.99996
Group 18	total	0.30	0.19	0.20	0.31	0.28	0.22	0.17	0.33	0.99994
	3'-UTR	0.30	0.19	0.20	0.31	0.28	0.22	0.17	0.33	1.00000
	5'-UTR	0.33	0.16	0.17	0.34	0.41	0.15	0.16	0.28	1.00000
	CDS	0.27	0.22	0.22	0.29	0.28	0.26	0.18	0.28	1.00000
	Intron	0.30	0.19	0.19	0.32	0.28	0.22	0.17	0.33	0.99995

Table S6 | The comparison of known motifs' enrichments in binding sites inferred by multiple methods.

Known motif	Method	Group/individual RBP	# of binding sites containing the known motif	# of total binding sites[1]	Percentage of binding sites containing the known motif	Odd ratio	p-value
UGUGUA	RBPgroup	Group 4	599	2,087	0.287		
	graphprot	HNRNPA1	105	2,087	0.050	5.704	4.12E-74
		HNRNPA2B1	107	2,087	0.051	5.597	3.59E-73
		HNRNPF	102	2,087	0.049	5.870	1.52E-75
		HNRNPH1	65	2,087	0.031	9.212	2.29E-96
		HNRNPM	203	2,087	0.097	2.950	2.39E-39
		HNRNPU	138	2,087	0.066	4.339	8.31E-60
	ideep	HNRNPA1	135	2,087	0.065	4.436	5.65E-61
		HNRNPA2B1	118	2,087	0.057	5.075	3.33E-68
		HNRNPF	98	2,087	0.047	6.109	1.69E-77
		HNRNPH1	77	2,087	0.037	7.776	6.63E-89
		HNRNPM	62	2,087	0.030	9.657	1.64E-98
		HNRNPU	99	2,087	0.047	6.048	5.27E-77
	ionmf	HNRNPA1	97	2,087	0.046	6.172	5.42E-78
		HNRNPA2B1	139	2,087	0.067	4.308	2.01E-59
		HNRNPF	144	2,087	0.069	4.159	9.64E-58
		HNRNPH1	107	2,087	0.051	5.597	3.59E-73
		HNRNPM	93	2,087	0.045	6.438	5.30E-80
		HNRNPU	150	2,087	0.072	3.992	1.50E-55
UGUA	RBPgroup	Group 5	1,045	1,716	0.609		
	graphprot	CPSF6	821	1,716	0.478	1.273	2.99E-05

		NUDT21	542	1,716	0.316	1.928	4.76E-26
	ideep	CPSF6	666	1,716	0.388	1.569	5.61E-14
		NUDT21	752	1,716	0.438	1.390	2.11E-08
	ionmf	CPSF6	750	1,716	0.437	1.393	1.75E-08
		NUDT21	359	1,716	0.209	2.910	1.41E-56
AAUAAA	RBPgroup	Group 6	3,528	7,646	0.461		
	graphprot	CSTF2	2,343	7,646	0.306	1.506	9.21E-40
		CSTF2T	2,274	7,646	0.297	1.551	5.15E-45
	ideep	CPSF6	2,606	7,646	0.341	1.354	2.26E-23
		NUDT21	2,576	7,646	0.337	1.370	5.28E-25
	ionmf	CPSF6	2,688	7,646	0.352	1.312	2.42E-19
		NUDT21	2,585	7,646	0.338	1.365	1.68E-24
AAUAAA	RBPgroup	Group 13	298	1,265	0.236		
	graphprot	ALKBH5	25	1,265	0.020	11.912	8.40E-55
		CPSF1	19	1,265	0.015	15.667	9.89E-60
		CPSF2	37	1,265	0.029	8.049	1.06E-46
		CPSF3	24	1,265	0.019	12.408	8.44E-56
		CPSF4	17	1,265	0.013	17.524	1.12E-61
	ideep	ALKBH5	31	1,265	0.025	9.607	1.09E-50
		CPSF1	17	989	0.017	13.699	2.09E-48
		CPSF2	43	1,105	0.039	6.050	1.05E-36
		CPSF3	29	1,265	0.023	10.269	4.97E-52
		CPSF4	27	1,265	0.021	11.030	2.10E-53
	ionmf	ALKBH5	56	1,254	0.045	5.273	2.90E-36

		CPSF1	52	1,265	0.041	5.727	1.89E-38
		CPSF2	47	1,077	0.044	5.395	2.15E-33
		CPSF3	54	1,265	0.043	5.515	1.65E-37
		CPSF4	52	1,050	0.050	4.754	4.15E-30
UGUA	RBPgroup	Group 15	2,433	2,950	0.825		
	graphprot	CPSF6	1,697	2,950	0.575	1.434	1.21E-18
		CPSF7	1,846	2,950	0.626	1.318	7.88E-12
	ideep	CPSF6	1,496	2,950	0.507	1.626	2.02E-31
		CPSF7	1,562	2,950	0.529	1.558	1.17E-26
	ionmf	CPSF6	1,455	2,950	0.493	1.672	1.36E-34
		CPSF7	1,342	2,950	0.455	1.813	1.57E-44

[1] We sampled the same number of total binding sequences based on top score of each method in order to make a fair comparison, except for a few cases in which some methods predicted two few binding sites.

We used 2/3 of the HEK293/HEK293T's CLIP-seq data as training data. For iONMF, we used k-mer (k = 4) frequency, RNA secondary structure (predicted by RNAfold) and genomic region features, but not gene ontology or RBP co-binding features. The RBP co-binding feature was not included because it will leak information of negative set (excluded binding sites of any RBP) and lead to ~100% AUC. For iDeep, the features are the same as iONMF except that RNAcompete motifs' occurrences were included in the feature set. For GraphProt, we extended the binding sites (upstream and downstream 150nt) and then marked the original binding sites as the viewpoints. For the training set, we selected up to 10,000 binding sites as positives and randomly sampled 10,000 sites not overlapping with any binding sites as negatives.

The remaining ~1/3 of the binding sites excluding the training set were used for test and evaluation. In our method, we defined group related binding sites as the binding site with values in the coefficient matrix above a threshold. For each RBP in RBP groups identified by our method, we defined binding sites for individual RBPs by one of the other three methods. Then, we scanned the known motifs in the group related binding

sites inferred by our method, as well as the individual RBPs' binding sites predicted by the other three methods. Then we compared the motif enrichment (fraction of binding sites that significantly match the known motifs) between our method and the other three methods. The odd ratio was calculated using four values: total number of group related binding sites defined by our method, individual RBP binding sites defined by other three methods, the number of binding sites containing known motif in group related binding sites, the number of binding sites containing known motif in individual RBP binding sites. The significance (p-value) was obtained from Fisher's exact test. As we expected, the known motifs were more enriched in the group related binding sites identified by our method than the binding sites of individual RBP identified by the other three methods.

Please note that these results were not based on direct comparisons because of different purposes of the methods. All the three published methods, GraphProt, iONMF and iDeep, aim to predict the binding sites of individual RBPs. However, none of these methods provides a utility to explicitly identify the binding sites/sequences associated to a group of RBPs.

Supplementary References

1. Zuberi K, Franz M, Rodriguez H, Montojo J, Lopes CT, Bader GD, Morris Q: **GeneMANIA prediction server 2013 update.** *Nucleic Acids Res* 2013, **41:**W115-122.
2. Memczak S, Jens M, Elefsinioti A, Torti F, Krueger J, Rybak A, Maier L, Mackowiak SD, Gregersen LH, Munschauer M, et al: **Circular RNAs are a large class of animal RNAs with regulatory potency.** *Nature* 2013, **495:**333-338.
3. Kishore S, Jaskiewicz L, Burger L, Hausser J, Khorshid M, Zavolan M: **A quantitative analysis of CLIP methods for identifying binding sites of RNA-binding proteins.** *Nat Methods* 2011, **8:**559-564.
4. Hafner M, Landthaler M, Burger L, Khorshid M, Hausser J, Berninger P, Rothballer A, Ascano M, Jr., Jungkamp AC, Munschauer M, et al: **Transcriptome-wide identification of RNA-binding protein and microRNA target sites by PAR-CLIP.** *Cell* 2010, **141:**129-141.
5. Baltz AG, Munschauer M, Schwanhausser B, Vasile A, Murakawa Y, Schueler M, Youngs N, Penfold-Brown D, Drew K, Milek M, et al: **The mRNA-bound proteome and its global occupancy profile on protein-coding transcripts.** *Mol Cell* 2012, **46:**674-690.
6. Martin G, Gruber AR, Keller W, Zavolan M: **Genome-wide analysis of pre-mRNA 3' end processing reveals a decisive role of human cleavage factor I in the regulation of 3' UTR length.** *Cell Rep* 2012, **1:**753-763.
7. Macias S, Plass M, Stajuda A, Michlewski G, Eyras E, Caceres JF: **DGCR8 HITS-CLIP reveals novel functions for the Microprocessor.** *Nat Struct Mol Biol* 2012, **19:**760-766.
8. Hoell JI, Larsson E, Runge S, Nusbaum JD, Duggimpudi S, Farazi TA, Hafner M, Borkhardt A, Sander C, Tuschl T: **RNA targets of wild-type and mutant FET family proteins.** *Nat Struct Mol Biol* 2011, **18:**1428-1431.
9. Kishore S, Gruber AR, Jedlinski DJ, Syed AP, Jorjani H, Zavolan M: **Insights into snoRNA biogenesis and processing from PAR-CLIP of snoRNA core proteins and small RNA sequencing.** *Genome Biol* 2013, **14:**R45.
10. Ascano M, Jr., Mukherjee N, Bandaru P, Miller JB, Nusbaum JD, Corcoran DL, Langlois C, Munschauer M, Dewell S, Hafner M, et al: **FMRP targets distinct mRNA sequence elements to regulate protein expression.** *Nature* 2012, **492:**382-386.
11. Huelga SC, Vu AQ, Arnold JD, Liang TY, Liu PP, Yan BY, Donohue JP, Shiue L, Hoon S, Brenner S, et al: **Integrative genome-wide analysis reveals cooperative regulation of alternative splicing by hnRNP proteins.** *Cell Rep* 2012, **1:**167-178.
12. Katz Y, Wang ET, Airoldi EM, Burge CB: **Analysis and design of RNA sequencing experiments for identifying isoform regulation.** *Nat*

Methods 2010, **7:**1009-1015.
13. Hafner M, Max KE, Bandaru P, Morozov P, Gerstberger S, Brown M, Molina H, Tuschl T: **Identification of mRNAs bound and regulated by human LIN28 proteins and molecular requirements for RNA recognition.** *RNA* 2013, **19:**613-626.
14. Sievers C, Schlumpf T, Sawarkar R, Comoglio F, Paro R: **Mixture models and wavelet transforms reveal high confidence RNA-protein interaction sites in MOV10 PAR-CLIP data.** *Nucleic Acids Res* 2012, **40:**e160.
15. Farazi TA, Leonhardt CS, Mukherjee N, Mihailovic A, Li S, Max KE, Meyer C, Yamaji M, Cekan P, Jacobs NC, et al: **Identification of the RNA recognition element of the RBPMS family of RNA-binding proteins and their transcriptome-wide mRNA targets.** *RNA* 2014, **20:**1090-1102.
